# Polymeric Nanoparticle Delivery of Combination Therapy with Synergistic Effects in Ovarian Cancer

**DOI:** 10.3390/nano11041048

**Published:** 2021-04-20

**Authors:** Shani L. Levit, Christina Tang

**Affiliations:** Chemical and Life Science Engineering Department, Virginia Commonwealth University, Richmond, VA 23284, USA; levitsl@vcu.edu

**Keywords:** polymer, drug delivery, cancer, combination chemotherapy, nanocarrier, therapeutic efficacy, ovarian carcinoma, synergy

## Abstract

Treatment of ovarian cancer is challenging due to late stage diagnosis, acquired drug resistance mechanisms, and systemic toxicity of chemotherapeutic agents. Combination chemotherapy has the potential to enhance treatment efficacy by activation of multiple downstream pathways to overcome drug resistance and reducing required dosages. Sequence of delivery and the dosing schedule can further enhance treatment efficacy. Formulation of drug combinations into nanoparticles can further enhance treatment efficacy. Due to their versatility, polymer-based nanoparticles are an especially promising tool for clinical translation of combination therapies with tunable dosing schedules. We review polymer nanoparticle (e.g., micelles, dendrimers, and lipid nanoparticles) carriers of drug combinations formulated to treat ovarian cancer. In particular, the focus on this review is combinations of platinum and taxane agents (commonly used first line treatments for ovarian cancer) combined with other small molecule therapeutic agents. In vitro and in vivo drug potency are discussed with a focus on quantifiable synergistic effects. The effect of drug sequence and dosing schedule is examined. Computational approaches as a tool to predict synergistic drug combinations and dosing schedules as a tool for future nanoparticle design are also briefly discussed.

## 1. Introduction

Ovarian cancer is primarily diagnosed in advanced stages (stage III and later) when the 5-year survival rate is only 30% [1,2,3,4,5]. The standard of care includes surgery to remove the majority of the tumor followed by chemotherapy [6,7,8]. At advanced stages, surgery alone is ineffective at completely removing the cancer as microscopic tumor tissue and macroscopic peritoneal implants form [9]. For these patients, platinum-based chemotherapy, such as cisplatin, following surgery was the standard of care for over 40 years. Platinum agents induced cytotoxicity by disrupting deoxyribonucleic acid (DNA) synthesis and normal cellular function. In the 1990’s the United States Food and Drug Administration approved paclitaxel (Taxol), an extract from the bark of the Yew tree for cancer treatment [6].

However, treating ovarian cancer remains challenging with the currently available chemotherapeutic agents [4,5]. Combination and sequential treatment schedules of drug combinations is a common practice for managing recurrent ovarian cancer [4,5,10,11]. For example, one of the common sequences is first-line carboplatin and paclitaxel therapy followed by a re-treatment of both drugs at first relapse. While this treatment has been found to prolong survival and improve quality of life for patients, it is associated with severe systemic toxicity and only a small population of patients exhibit long-term remission [9].

Due to various factors such as the type of ovarian cancer, genetic mutations, and development of resistance mechanisms, selection of appropriate drug combinations as well as treatment schedules is challenging. Both clinical and pre-clinical studies have investigated sequence schedules of drug combinations to overcome these limitations. The goal is to leverage the downstream effects of the drugs to induce synergistic interactions [10,12,13]. Clinically, sequences of drug combinations are treated on the order of days to weeks [14]. In contrast, preclinical studies performed in in vitro and in vivo animal studies of sequential drug treatments are often conducted on the order of hours i.e., the same timescale as many drug-activated pathways [15,16,17]. Therefore, direct comparisons between clinical and pre-clinical results are challenging due to a difference in time schedules. Furthermore, while delivering therapeutic dosages on the same time scale as cellular activity can enhance therapeutic efficacy, clinical translation of the protocols is difficult. Two major challenges for clinical translation are patient compliance and increased cytotoxicity. Thus, treating patients with recurrent ovarian cancer and acquired drug resistance mechanisms remains a significant challenge [4,5,9].

Nanoparticle delivery of drug combinations can overcome many of these limitations. Encapsulating drugs into nanocarriers allows for control over the pharmacokinetic properties by controlling drug release as well as increasing circulation half-life and lowering interaction with healthy tissue. Nanoparticle carriers can be designed to accumulate in tumor tissue by utilizing the enhanced permeability and retention (EPR) effect. Additionally, several nanoparticle formulations loaded with chemotherapeutic drugs have been approved by the United States Food and Drug Administration for treating ovarian cancer [4,5,18]. Specifically, polymer nanoparticles offer control over parameters including size and material selection i.e., drug combinations. Therefore, polymer nanoparticles are an especially attractive approach for improving delivery of chemotherapeutic agents [18,19]. With appropriate selection of the polymer, nanocarriers can facilitate high loading of hydrophobic drugs and tunable release of the payload. The chemical-physical properties can also be potentially tuned to ensure stability during storage and administration [20]. Furthermore, use of biodegradable polymers is promising to avoid accumulation of the nanocarriers. Biodegradable polyesters such as poly (lactic acid) (PLA), poly (lactic acid-co-glycolic acid) (PLGA) and poly (ε-caprolactone) are especially promising due to their well-established use [20]. PLGA is a particularly promising system to tune the degradation rate of the system [18,21]. A formulation of paclitaxel using PLA has entered Phase II clinical trials [20,22].

We review polymer nanoparticle (e.g., micelles, dendrimers, and lipid nanoparticles) carriers of drug combinations formulated to treat ovarian cancer. In particular, the focus of this review is nanoparticle formulations of at least two small molecule therapeutic agents. To be reviewed, the (1) formulations were assessed using ovarian cancer cell line(s) and (2) drug potency and/or quantifiable measures of synergistic drug interactions in vitro or in vivo were included. Section 3 has been organized by nanocarrier type i.e., micelles/nanoparticles, polymer/lipid hybrid nanoparticles, and dendrimers. Where appropriate, the subsections are further subdivided by drug type i.e., platinum-based drug combination, taxol-based drug combination, etc.

## 2. Background

### 2.1. Drug Resistance Mechanisms

**Figure 1:**
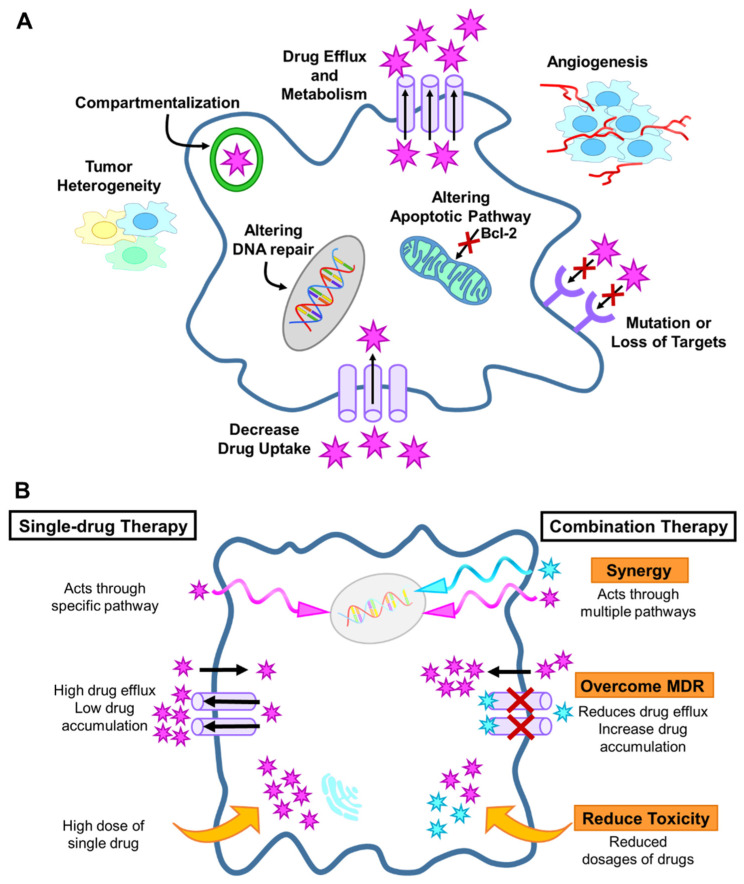
(**A**) Overview of mechanisms affecting cancer cell resistance to anticancer therapy ranging from changing protein expression to effecting drug accumulation, drug metabolism, to repair of apoptotic pathways. (**B**) Advantages of drug combination for treating ovarian cancer. Synergy can be observed when the drug combinations act through multiple pathways. Combinations can overcome multi-drug resistance (MDR) mechanisms to increase anticancer activity. Delivery of drug combinations can also reduce toxicity by reducing the required doses of each drug.

With chemotherapy treatments, many patients relapse due to development of drug resistance mechanisms. Drug resistance is attributed to several factors: drug inactivation by compartmentalization or detoxification, drug efflux, mutation or loss of drug targets, DNA repair, cell death inhibition, or alteration of apoptotic pathways. Additional factors can include tumor heterogeneity as well as angiogenesis [11,23]. Ovarian cancer cells are known to become resistant to a variety of drugs including cisplatin, carboplatin, and paclitaxel (Figure 1A) [17].

One of the main contributors to drug resistance to paclitaxel is drug efflux, which results in decreased accumulation. Specifically, upregulation of ABC (adenosine triphosphate binding cassette) transporters, membrane proteins that regulate transport of substances across the cell membrane, increases drug efflux. This mechanism affects taxane agents but is not associated with low accumulation of platinum chemotherapeutics [11,17,23].

For platinum agents, detoxification or inhibition of the anticancer activity of drug reduces treatment efficacy [23]. For example, metallothionein and glutathione are known to play a role in detoxification either by altering drug transportation, enzymatic-catalysis of inactivation of platinum, or inhibiting DNA damage induced by platinum therapies [17]. Drug resistance for platinum agents is also affected by the rate of DNA repair [17]. Drug resistance can also occur by cell inhibition of apoptosis and autophagy pathways. For example, apoptosis can be inhibited by overexpression of anti-apoptotic proteins such as B-cell lymphoma 2 (Bcl-2) and Protein kinase B (Akt) [17]. More detailed reviews of drug resistance mechanism can be found elsewhere [17,23].

Overall, drug resistance to first line treatments such a paclitaxel and platinum agents makes treating ovarian cancer a challenge. To improve ovarian cancer patient outcomes, several treatment approaches have been attempted. Many of these strategies focus on overcoming or preventing multidrug resistance. These approaches include dose-intense chemotherapy, intraperitoneal administration, and combination therapy. Dose-intense chemotherapy has also shown limited efficacy with the maximum dose limitations in clinical settings. Several studies found that weekly infusions of paclitaxel alone or with carboplatin improved survival with minimal progression. However, there was cumulative toxicity with multiple treatments. Intraperitoneal drug administration intends to expose cancer cells to higher drug concentration at the tumor site and has also been investigated in ovarian cancer. Survival time improves, but severe toxicity was observed [24].

Combination therapy provides several advantages including reducing drug dosage to lower toxicity, inhibiting cancer activity with multiple molecular targets, and overcoming drug resistance mechanisms (Figure 1B) [25]. Currently, the standard of care is a two-drug combination treatment of platinum agent with paclitaxel. Some treatment methods have shifted to using less toxic platinum agents to reduce toxicity (e.g., carboplatin rather than cisplatin) [14,24].

To further improve the treatment efficacy of drug combinations, the effect of dosing schedules on drug resistance, relapse, and toxicity have been considered. For example, there is evidence to suggest that platinum-free intervals improve patient response by reducing tumor resistance to therapies [26]. Sequential treatment schedules have been examined in clinical trials for both previously untreated and recurrent ovarian cancer [10,26]. For example, a phase II study of sequential treatment of carboplatin followed by paclitaxel and then topotecan over the course of 150 days showed this treatment has a high response rate and prolonged survival time compared to a matched group that was treated with carboplatin [27]. These studies show promising results for further investigation of sequential drug delivery. 

Another approach to improve the efficacy of drug combinations has been nanoparticle formulations [4,14,28,29,30,31,32]. Nanoscale encapsulation of chemotherapeutics such as doxorubicin increased drug efficacy and reduced cardiotoxicity compared to the conventional free drug formulation and was approved by the United States Food and Drug Administration in 1995 [18]. The next generation of focused on overcoming biological barriers of the circulatory system. Specifically, these materials are formulated to leverage EPR effect avoiding nanoparticle clearance by opsonization. According to the EPR effect, nanoparticles delivered intravenously (IV) preferentially accumulate at the site of diseased tissue (e.g., solid tumors) due to its leaky vascular. Use of polymer particles e.g., PEGylation is advantageous. PEGylation, enhances drug stability in vivo by preventing enzymatic attack and recognition by the immune system, which increases circulation time. This increased circulation time is especially important for passive targeting to disease sites using the EPR effect. Additionally, nanoparticles can reduce systemic toxicity by increasing potency so that lower doses can be used [4,18,32]. Specifically, PEGylated liposomal doxorubicin (Doxil), has been shown to enhance drug efficacy in recurring or resistant ovarian cancers alone and in combination with other chemotherapeutic agents [26].

### 2.2. Quantification of Drug Interaction and Synergy

With the growing interest in drug combination therapies and drug screening studies, it is important to understand drug interactions. Drug interactions are affected by the drug targets, activated downstream pathways, and other intracellular biochemical interactions. Drug interactions have a positive or negative effect on treatment efficacy. If there is no cross-interaction the combination will have an additive effect. If the interaction increases treatment efficacy and has a greater overall effect than the sum of the individual drugs, then the combination is considered synergistic. However, when the combination of multiple drugs produces a smaller effect than the sum of the individual components, the drug interaction is antagonistic [33,34].

Investigating and quantifying drug interactions are particularly important for developing new drug combinations and dosing schedules to enhance treatment efficacy (i.e., to achieve synergistic effects). There are several methods for quantifying drug interactions. For example, the Bliss independence model is based probability theory assuming the drugs have no interactions [35]. The Loewe additivity model also describes drug interactions. This model assumes that a drug cannot interact with itself and that two doses from two different compounds produce the same effect therefore they can be substituted. The second assumption accounts for similar mechanisms of action of the two compounds [35,36]. In practice, empirical analyses are more common. Specifically, the isobologram and combination index are two common approaches for quantifying the synergistic activity of anticancer drugs [33,34].

**Figure 2:**
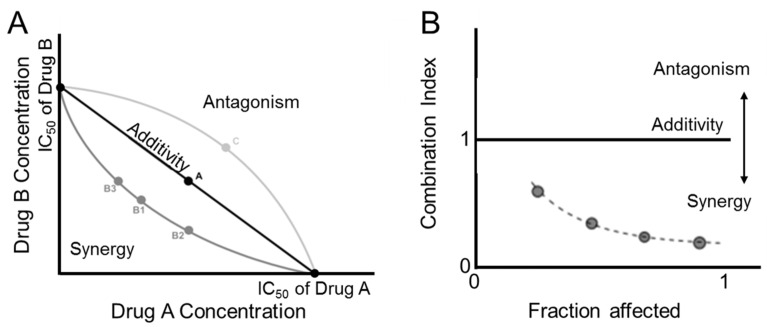
(**A**) Isobologram to visualize effect of combining drug A and B. The line between the IC50 of drug A and B indicates an additive effect. Below the line of additivity indicates synergistic drug interactions; above the line of additivity indicates antagonistic drug interactions. (**B**) Visual representation the combination index (*CI*) versus fraction affected (f_A_) Figure adapted from [37], Copyright © 2012 Breitinger. Licensee IntechOpen.

The isobologram is an approach to visualizing drug interactions (Figure 2A). The plot is based on the interaction index (Equation (1)) calculated as:

(1) $$I=\frac{D_{1}}{ID_{X,1}}+\frac{D_{2}}{ID_{X,2}}$$

where *D*_1_ and *D*_2_ are the concentrations of drug 1 and 2 delivered in combination and *ID_X,_*_1_ and *ID_X_*_,2_ are the drug concentrations that produce the same level of effect, *X*, when treated alone. Often it is described at 50% inhibition (*X* = 50%). The interaction index equals a value of one when there is no drug interaction and the effect is additive. This is represented in the isobologram as the line of additivity. Interaction indices below 1 indicate synergistic drug interactions and fall below the line of additivity. In contrast, interaction indices above 1 indicate antagonist drug interactions and fall above the line of additivity [34,37].

A similar approach uses the combination index to determine synergistic, additive, or antagonistic drug interactions. This approach is based on the median effect analysis of the dose response curves was derived by Chou et al. [33,37]. The combination index (CI) for multiple drugs is defined by Equation (2):

(2) $$CI=\overset{n}{\underset{j=1}{\sum }}\frac{{\left(D\right)}_{j}}{{\left(ID_{x}\right)}_{j}}$$

where *n* is the number of drugs in the combination. When the *CI* is equal to 1 the effect is additive. *CI* values less than 1 indicate the effect is synergistic and values above 1 indicate the effect is antagonistic (Figure 2B).

It should be noted that the drug ratios and percent inhibition used can affect the drug interaction results [33,34,37]. Furthermore, since the dose-response curve is non-linear there can be a concentration range in which the drugs are synergistic or antagonistic [33,34,37]. In the case of three or more drugs and varying schedules of delivery, determining synergistic interaction can be more complex [37]. Overall, characterizing the synergy of drug combinations can be a useful tool for screening drug combinations.

### 2.3. Sequence-Dependent Synergy of Free Chemotherapeutic Drugs

Overall, clinical practices are shifting to sequential chemotherapy of drug combinations (e.g., platinum-free intervals). To understand the effect of drug combinations on multidrug resistance, there has also been an increase in preclinical studies investigating drug combinations as well as dose schedules. This approach is promising for ovarian cancer because it could prevent cross-resistant therapies, minimize cumulative toxicities, as well as induce synergistic drug interactions [28]. We begin by examining sequential delivery of free drug (small molecule) combinations. Specifically, we focus on platinum and taxane (first line treatments) based combinations [14,25,38].

#### 2.3.1. Platinum Based Combinations

Platinum and taxane agents are often paired. Both have cell cycle-dependent effects [14]. Sequential delivery could facilitate cell cycle arrest at specific phases to increase apoptosis, leading to cancer cell death [15]. The interaction of these drugs in combination has been examined in ovarian cancer cell models to evaluate the effect of sequential delivery on drug synergy. For example, sequential delivery of cisplatin with taxane agents has been evaluated on both platinum-resistant and-sensitive cells with cell-dependent outcomes [39,40]. The efficacy of the drug combination (synergistic vs. antagonistic) can be sequence dependent. Jekunen et al. observed synergistic drug interactions (*CI* ~ 0.1) when cells (platinum sensitive and platinum resistant A2780) were exposed to Taxol for 19 h followed by concurrent exposure to Taxol and cisplatin for 1 h. However, reversing this sequence was antagonistic rather than synergistic [40]. Judson et al. investigated combination of cisplatin with paclitaxel in platinum-resistant and-sensitive cell lines (A2780, A2780CP, OV-2008, SKOV-3, OVC-420, OVCA-429, OC-194, OC-494). Paclitaxel alone induced apoptosis in both cisplatin-resistant and-sensitive cell lines. However, the addition of cisplatin inhibited paclitaxel-induced apoptosis in cisplatin-resistant cells. Similar effects were also observed when the drugs were sequentially administered with paclitaxel followed by cisplatin. The results suggest that cisplatin targets are downstream of the primary targets of paclitaxel in cisplatin-resistant cells (since the cisplatin did not inhibit stabilization of the microtubules nor the expression of Bcl-2) [41].

Vanhoefer et al. performed a similar study examining the sequential delivery of paclitaxel and cisplatin on ovarian adenocarcinoma cells (EOVI and EOV2) from patients pretreated with platinum therapies using isobologram analysis. Treating the cells with paclitaxel 24 h prior to cisplatin produced a synergistic effect. However, when the two drugs were administered simultaneously or in the reverse sequence, the treatment was antagonistic. Furthermore, pre-exposure to cisplatin resulted in long-lasting antagonistic effects due to decreased intracellular accumulation of paclitaxel, downregulation of glutathione inhibiting cytotoxicity, and delayed cell phase transition from S-phase to G_2_/M phase. Overall, the findings found a schedule-dependent synergy of cisplatin plus paclitaxel [42].

The effect of the sequence is drug combination dependent. Second generation platinum therapies (e.g., carboplatin) have also been considered (due to the relatively high systemic toxicity of cisplatin) in combination with taxane drugs to determine the synergistic effect and an optimal sequencing schedule [43,44]. Some studies have observed sequential treatment of carboplatin followed by paclitaxel produced a synergistic effect, while reverse sequence and simultaneous treatment produced an additive effect using isobologram analysis [17].

**Figure 3:**
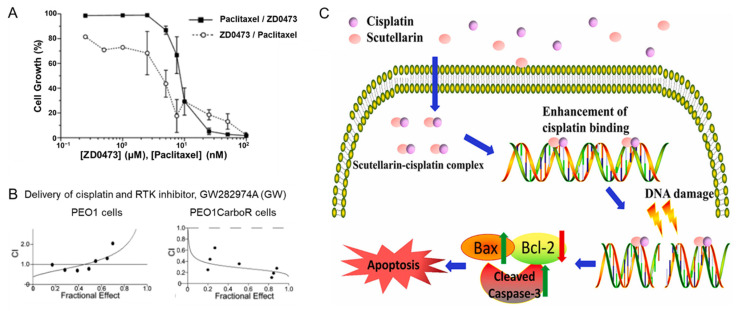
(**A**) Dose-response curve shows sequence-dependent response of sequentially delivering a platinum drug, ZD0473 and paclitaxel in in platinum resistant cells, A2780cis. The results show that delivering platinum drug followed by paclitaxel exhibits greater reduction in cell growth. Reprinted from [45], Copyright 2002, with permission from Elsevier. (**B**) Combination index analysis of cisplatin and a RTK inhibitor, GW282974A (GW), comparing PEO1 and platinum-resistant cells, PEO1CarboR cells showing synergy (*CI* < 1) depends on drug concentration and cell type Reprinted from [46], Copyright 2006, with permission from Elsevier. (**C**) Overview of enhanced apoptosis in ovarian cancer cells due to combination treatment of scutellarin and cisplatin. Specifically, the combination treatment increases the ability of cisplatin to bind to DNA resulting in increased the level of cleaved capsase-3 and increased the ratio of Bax/Bcl-2 which promote apoptosis. Overall the result of the drug combination is synergistic (*CI* 0.566–0.796 depending on drug ratio) Reprinted from [47]. Copyright 2019, with permission from Elsevier.

Additional platinum-based agents in combination with paclitaxel have also been considered. For example, a new generation platinum therapeutic agent ZD0473 was studied in combination with paclitaxel in vitro. Four different human ovarian carcinoma cells (A2780, A2780cis, CH1, A2780/E6) were examined with and without platinum resistance. In all the cell lines studied, simultaneous treatment with ZD0473 and paclitaxel produced a synergistic effect as indicated by the combination index. ZD0473 administered 24 h prior to paclitaxel generally produced a synergistic effect compared to the reverse sequence (Figure 3A). Combining ZD0473 with other chemotherapeutics did not have synergistic effects. For example, with doxorubicin there was either antagonistic effect or no drug interaction in all cell lines. The difference in the cell response to the drug combinations can be attributed to a difference in drug resistance mechanism and protein expression such as p53 [45].

As an alternative to taxane, platinum therapies have also been used in combination with inhibitory agents to treat ovarian cancer. These agents target specific proteins and genes to overcome drug resistance mechanisms, overexpression of angiogenic or anti-apoptotic genes. Examples include: include tyrosine kinase inhibitors, Poly-ADP ribose polymerase (PARP) inhibitors, and Akt inhibitors (Figure 3B) [23,24,48]. When used in combination, sequence-dependent synergistic effects have been observed [49,50]. For example, a proteasome inhibitor, bortezomib, was studied in combination with carboplatin, oxaliplatin, or [*trans*-bis (3-hydroxypyridine) dichloroplatinum(II)]. Platinum-sensitive (A2780 and SKOV-3) and resistant (A2780cis and A2780/ZD0473R) cells were selected for these in vitro experiments. The results found that bortezomib enhanced intracellular accumulation of the platinum drug which increased platinum-induced down-regulation of copper transporter 1. An antagonistic effect was observed when the platinum agent was delivered prior to bortezomib in platinum-resistant cells (Figure 3C) [49]. These results demonstrate that chemotherapeutic agents can be paired with drugs that target overexpressed genes for cell specific therapy.

Platinum-based therapies have also been used in combination with natural compounds. For example, the effect of sequence on the efficacy of combinations of cisplatin with epigallocatechin-3-gallate (EGCG) was examined. Higher drug synergy (i.e., lower combination indices) were generally observed when cisplatin was administered first. Increasing the time between doses from 4 h to 24 h also increased synergy in both platinum-sensitive (A2780) and platinum-resistant cells (A2780cis). By delivering an antioxidant agent 4 h after cisplatin, the antioxidants can scavenge free radicals and target pro-apoptotic proteins to allow for greater platinum-DNA binding resulting in greater drug efficacy [51]. Similar effects using other natural compounds have also been reported [52]. Cisplatin has also been used in combination with natural compounds such as scutellarin, a flavonoid. Scutellarin has increased the cytotoxic effect of cisplatin in various ovarian cancer cell lines, specifically OVCAR-3 and SKOV-3. In SKOV-3 cells, the combination index was between 0.566 and 0.796 depending on the drug ratio indicating a synergistic effect. The platinum accumulation was comparable for cells treated with cisplatin compared to the combination. Interestingly, the formation of Pt-DNA adducts was higher for the combination treatment than treatment with cisplatin alone. Further analysis indicated that the combination treatment the level of cleaved capsase-3 and increased the ratio of Bax/Bcl-2 compared to cisplatin alone, which promote apoptosis [47].

Taken together, these studies demonstrate that sequence dependent synergy depends on many factors including (but not limited to): cell type, drug combination, drug ratio, and treatment schedule. The relation of the drug targets to the downstream pathway of platinum therapies is also an important consideration. Upregulation of pro-cancer proliferating pathways or formation of free radicals can also enhance drug efficacy.

#### 2.3.2. Taxane Based Combinations

Taxane agents (e.g., paclitaxel) are another class of chemotherapeutic commonly used to treat ovarian cancer [14,25,38]. Paclitaxel targets microtubules in the cell and inhibits polymerization necessary for mitosis preventing proliferation and eventually leading to cell death [53,54]. Drug resistance mechanisms, high systemic toxicity, and poor bioavailability are major limitations of paclitaxel efficacy. Therefore, paclitaxel is often paired with other drug to improve therapeutic efficacy and patient outcomes [14,55,56]. For example, paclitaxel has been combined with bleomycin. Using isobologram analysis, drug synergy was observed in HEY cells [13]. Sepantronium bromide (YM155), an inhibitor of survivin, binds directed to the C-terminus of ribonucleic acid (RNA) binding proteins [57]. It has been combined with docetaxel to treat various ovarian cancer cell lines in vitro (A2780, Taxol sensitive and Taxol resistant). YM155 had a synergistic effect with docetaxel (*CI* < 1) in both cell lines. Paclitaxel has also been combined with zibotentan (ZD4054), an endothelin-1 and endothelin A receptor (ETAR) antagonist (overexpression of ETAR is associated with ovarian carcinomas). The combination of ZD4054 with paclitaxel significantly inhibited HEY cell proliferation in vitro. The results were further improved with the co-treatment with cisplatin. The combination of all three drugs also inhibited tumor growth and neovascularization in vivo (HEY xenograft). These results support the concept that drug combinations with different target mechanisms can result in synergistic effects [58]. Paclitaxel has also been combined with cannabidiol with synergistic effects as indicated by the combination index between 0.7 and 1 in SKOV-3 cells in vitro. Pre-administration with cannabidiol could be used to reduce the required dose of paclitaxel necessary to achieve the same effect on cell viability [59]. This result demonstrates sequence and dosing schedule can impact treatment efficacy.

In cancer, epidermal growth factor receptor (EGFR) or its downstream proteins are often overexpressed which promotes tumor growth. Therefore, paclitaxel is often paired with inhibitors to inhibit pro-tumorigenic cell function [23]. For example, CRM197, a class of heparin-binding epidermal growth factor-like growth factor (HB-EGF) inhibitor drugs plays a role in inactivating extracellular related protein kinase and Akt to overcome paclitaxel drug resistance. CRM197 has been sequentially treated with paclitaxel to improve drug efficacy (reduced tumor volume) in 2D cell models (SKOV-3 cells) and SKOV-3 mouse models overexpressing HB-EGF [60]. Other inhibitors include kinase inhibitors. For example, flavopiridol is a first cyclin-dependent kinase inhibitor involved in cell cycle regulation and has been studied in combination with paclitaxel. Specifically, sequential combinations of paclitaxel and flavopiridol were examined in SKOV-3 cells. Sequential delivery of Taxol (treated for 24 h) followed by flavopiridol (for 24 h) produced the greatest cell death and highest apoptotic rate in vitro compared to single drug treatment [61].

These drug combinations can also be administered with the aim of using various treatment schedules to increase treatment efficacy and/or reduce toxicity. For example, the serine/threonine kinase, Akt, plays a prominent role in promoting cell survival and inhibiting apoptosis; therefore, inhibition of this protein is an important factor in promoting cytotoxicity of cancer therapies [23]. Another study investigated an Akt inhibitor (MK-2206), downstream of EGFR, in combination with paclitaxel. The combination treatment of MK-2206 and paclitaxel produced a synergistic interaction (*CI* 0.07) in ovarian cancer cells (SKOV-3) due to suppression of both Akt and EGFR-2 signaling pathways in vitro and in vivo [62]. MK-2206 was also synergistic with a variety of other chemotherapeutic drugs including doxorubicin (topoisomerase inhibitor), camptothecin (topoisomerase inhibitor), gemcitabine (anti-metabolite), 5-FU (anti-metabolite), and carboplatin (DNA cross-linker) in A2780 ovarian cancer cells [62].

Another class are histone deacetylase and histone acetyl transferases which are commonly used as anticancer targets because of their role in gene transcription. Histone deacetylase inhibitors have a role in arresting growth, promoting apoptosis, and regulating the cell cycle (p21) by increasing expression of pro-apoptotic proteins while decreasing anti-apoptotic proteins. Inhibitors of these targets can arrest tumor growth and induce apoptosis [63]. A study by Modesitt and Parsons examined sequential treatment of vorinostat, a histone deacetylase inhibitor, with paclitaxel on three different cell types (SKOV-3, OVCAR-3, MDAH-2774) using a murine model. Treatment of paclitaxel was followed by a low dose of vorinostat increased cytotoxicity when using the MDAH-2774 cells whereas the sequence did not affect the other cell types. The MDAH-2774 cells were thought to be susceptible to treatment with vorinostat due to their relatively low expression of survivin and p21. However, the in vivo results did not show a difference between the sequences of administration. The study concludes that the benefits of sequential and combination treatments are cell-dependent; therefore, the treatment needs to be tailored to specific ovarian cancer mutation to optimize therapeutic effects [64].

Alternatively, cyclooxygenase (COX) enzymes play a role in cell migration and tumorigenesis and have also been targeted with COX inhibitors to treat ovarian cancer [65]. Li et al. studied two COX inhibitors, celecoxib and SC-560 alone and in combination with paclitaxel, on SKOV-3 carcinoma cells xenografts. Alone, celecoxib and SC-560 significantly decreased tumor volume. The decrease in tumor volume was further enhanced by the addition of paclitaxel. The greatest decrease in tumor volume was observed in three-drug combination of both COX inhibitors and paclitaxel. There is also a decrease in cyclin D1 expression which can inhibit the cell cycle progression from G_1_ to S phase and affect the efficacy of paclitaxel [66].

Another class of drugs that have been used in combination with paclitaxel are metabolic regulating drugs. For example, the effects of sequential treatment with paclitaxel and 8-Chloro-adenosine 3′,5′-Monophosphate (8-Cl-cAMP), an antimetabolite, has been considered. Using the Chou-Talalay method, they examined the combination index (CI) while maintaining the same drug ratio. Overall, paclitaxel treatment before 8-Cl-cAMP was the most effective method with highest synergy (lowest CI). The drug synergy may be attributed to the activity of 8-Cl-cAMP which like paclitaxel, accumulates the cells in the G_2_-M phase of the cell cycle and inactivates stathmin increasing the proportion of stabilized tubulin which can be exposed to paclitaxel [67].

Another metabolic drug, lonidamine (inhibits aerobic glycolysis) has also been used to treat ovarian cancer in combination with paclitaxel. Orlandi et al., treated A2780 cells with sequential combinations of lonidamine and Taxol. They found that the efficacy of the treatment was sequence dependent. Specifically, synergy was observed when the cells were treated with Taxol prior to lonidamine. The reverse sequence or simultaneous delivery was antagonistic. Lonidamine was determined to not modify the effects induced by Taxol (cell cycle arrest, tubulin polymerization, and apoptosis) and instead impacted the induction of the Bax protein as well as other targets [68].

Synergistic drug interactions have been observed with sequential delivery of paclitaxel and various small molecule drugs. In many cases, delivery of paclitaxel prior to the secondary drug agent enhances treatment efficacy but depends on the drug combination and cell type. Selection of the appropriate drug pair to combine with paclitaxel is cell dependent and considerations include expression of key proteins downstream of the taxane target, intracellular drug accumulation, or enhancement of cell cycle arrest.

## 3. Nanoparticle Formulation of Drug Combinations

Overall, platinum and taxane based drug combinations have been well studied for treating ovarian cancer. Dosing schedules can be used to improve treatment efficacy. However, it is challenging to translate pre-clinical studies on sequential drug dosing (hours to days) to clinical (days to weeks) studies due to timescale disparity. Furthermore, there are many limitations of free drug formulations. For example, free drug formulation is limited by high systemic toxicity and poorly water-solubility [29,32]. Thus, achieving a safe and efficacious drug dose is a significant challenge [7].

**Figure 4:**
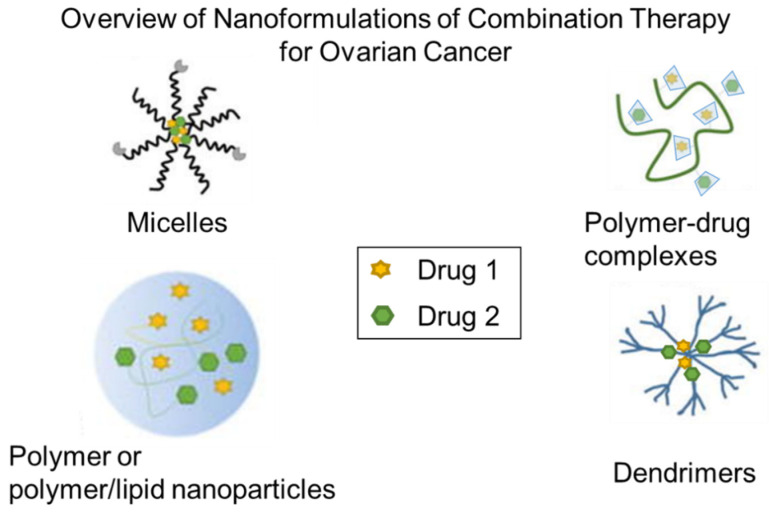
Schematic overview of the polymer-based nanocarriers used for combination therapy in ovarian cancer included in this review.

Formulation of these drugs combinations into nanoparticles for ovarian cancer treatment can address many of these challenges [29,32]. Incorporation of the drugs into nanoparticles can improve the solubility and reduce toxicity [18,69]. Controlled drug release from nanoparticles could facilitate sequential drug delivery to facilitate improved control of the pharmacokinetics [30,31,70]. Thus, we review polymer-based nanoparticles (micelles, dendrimers, and solid lipid polymer-based platforms (Figure 4)) for simultaneous and sequential delivery of platinum- or taxane-based combinations applied to ovarian cancer with an emphasis on quantitative evaluation of nanoparticle formulation on drug synergy.

### 3.1. Polymer Nanoparticles and Micelles

Micelles and amphiphilic (hydrophobic core/hydrophilic shell) polymer nanoparticles are advantageous for chemotherapeutic treatment because they can facilitate high drug loading, as well as controlled and stimuli-responsive drug release [30,69]. The formulation of polymeric micelles leverages the self-assembly of the amphiphilic polymer. Some of these self-assembly methods include ultrasonication, thin-film dispersion, and nanoprecipitation, where amphiphilic polymers form core-shell structures. Drugs are loaded into micelles either by encapsulation into the core or conjugation to the polymers. The selection of the polymer and ratio of the polymer to core materials affect the surface chemistry, degradation rate, as well as particle size and shape (important for the EPR effect i.e., selective accumulation at the tumor site) [18,71]. Thorough reviews of micellar nanoparticles for chemotherapeutic treatment of cancer can be found elsewhere [70,72]. In this review, we will focus on nanoparticles encapsulating platinum or taxane agents in combination with small molecules drugs for the treatment of ovarian cancer.

### 3.2. Platinum Based Combinations

Polymer nanoparticles encapsulating platinum-drug combinations have been investigated for ovarian cancer treatment (summarized in Table 1). This class of drug is commonly combined with paclitaxel for synergistic drug interactions. For example, carboplatin and paclitaxel were co-encapsulated into folic acid targeted PEGylated nanoparticles by click chemistry and sonication. Specifically, azide functionalized p-phosphonated calix[4]arene was used as a surfactant to stabilize paclitaxel and carboplatin nanoparticles formed by sonication. Folic acid-PEG-alkyne was conjugated to the azide functionalized p-phosphonated calix[4]arene nanoparticles using azide-alkyne click chemistry. The resulting particles had a hydrodynamic diameter of 160–185 nm by dynamic light scattering with a polydispersity index between 0.24 and 0.27. The drug ratio was fixed at 1:0.2 paclitaxel: carboplatin. Encapsulating the drugs into nanoparticles increased their potency in SKOV-3 and HO-8910 ovarian cancer cell lines as indicated by the~2-fold decrease in IC50 concentration compared to the free drug in the same ratio. Conjugating the nanoparticle to the folic acid-PEG further increased the potency in vitro. An increase in cell apoptosis by flow cytometry was also observed upon encapsulation. Encapsulation increased the cell mortality rate of SKOV-3 by 2.5-fold; conjugation further increased the cell mortality rate by 3-fold in vitro. The nanoparticle efficacy was also studied in vivo using ovarian cancer xenografts. Briefly, 5–6-week-old BALB/c mic were injected with SKOV-3 tumor cells. When the tumors reached a volume of 200 mm^3^, they were treated via an intratumor injection once every other day. The efficacy of the nanoparticle formulations was compared to the free drugs in solution. The conjugated nanoparticles significantly reduced the tumor volume after 18 days compared to the free drug and the nanoparticles without folic acid-PEG [73].

Co-loaded cisplatin, paclitaxel micelles have also been formulated into injectable hydrogels for ovarian cancer treatment for sustained, localized drug release. Shen et al. covalently linked to diblock copolymers of poly(ethylene glycol) and poly(lactide/glycolide) (mPEG-b-PLGA) copolymers to Pt(IV) prodrug. The prodrug was an amphiphilic stabilizer for the micelle; cisplatin was released upon intracellular reduction. It self-assembled into micelles, encapsulating paclitaxel. Its concentrated solution shows a reversible sol-gel transition as the temperature increases. The formulations were prepared by dissolving the paclitaxel and prodrug stabilizer in acetone, removing the solvent, freeze drying, and redissolving the mixture in water. The in vitro cytotoxicity was evaluated using SKOV-3 cells. The co-loaded formulation was significantly more potent than prodrug stabilizer as indicated by the over 3600-fold decrease in IC50 value. The drug combination was synergistic; the combination index was approximately 0.9 [74].

Alternative stabilizers such as peptide-based materials have also been considered. For example, cisplatin and paclitaxel were coloaded into polypeptide-based polymeric micelles. Specifically, triblock copolymers of PEG, glutamic acid, and phenylalanine were self-assembled into micelles with a hydrophobic phenylalanine core, intermediate glutamic acid shell, and PEG corona. The intermediate glutamic acid shell was crosslinked with carbodiimide chemistry. Paclitaxel and cis-dichlorodiamminoplatinum (II) were coloaded into the crosslinked micelles; paclitaxel was loaded into the hydrophobic core whereas the cisplatin coordinated with the carboxylic acids of the intermediate glutamic acid shell. Folic acid was conjugated to the drug loaded nanoparticles using PEG spacers (Fmoc-NH-PEG-NH_2_, molecular weight 7500 g/mol) via carbodiimide chemistry. The resulting crosslinked micelles were about 90 nm in diameter by dynamic light scattering. The efficacy of the nanoparticles was evaluated in vitro using A2780 ovarian cancer cells. Interestingly, conjugation of folic acid increased the potency of dual drug loaded nanoparticle as indicated by the 2-fold decrease in IC50 after 24 h. The in vivo efficacy was studied using peritoneal carcinomatosis generated by intraperitoneal injection of A2780/Luc cells treated every 4 days via tail vein injections. CA-125 a protein elevated in advanced ovarian cancer and was used as an indicator of tumor progression. Formulating the cisplatin into nanoparticles with paclitaxel reduced CA-125 levels compared to free cisplatin. Conjugating the nanoparticles to folic acid further reduced CA 125-levels. Conjugation to folic acid also significantly increased cisplatin accumulation in tumor tissue compared to formulations without folic acid. Finally, the co-loaded particles demonstrated improved tumor inhibition and survival compared to single drug formulations and the same drug ratio confirming the advantage of delivering the drug combination in a single carrier [75].

Wan et al. performed a similar study using amphiphilic triblock copolymer poly(2-methyl-2-oxazoline-*block*-2-butyl-2-oxazoline-*block*-2-methyl-2-oxazoline) micelles. The drug (paclitaxel and cisplatin with aliphatic tails (C_6_, C_8_ or C_10_)) loaded micelles were prepared by the thin film method. The drugs and block copolymer were dissolved in ethanol, the ethanol was evaporated, then the thin film was rehydrated in saline. The non-incorporated drug was removed by centrifugation. The resulting nanoparticles were between 35 and 150 nm in diameter depending on the prodrug and drug ratio. The potency of the formulations was evaluated in vitro using A2780, a cisplatin-sensitive, cell line. The most hydrophobic prodrug was approximately 30 times more potent than cisplatin as indicated by the decrease in IC50. The co-loaded micelle was more potent than the single-drug-loaded micelle. The effect (combination index) was highly dependent on the drug ratio; high ratios of paclitaxel to cisplatin prodrug were more likely to have synergistic effects. For example, 40:3 ratios of PTX to C6CP resulted in combination indexes less than 0.2 in A2780CisR cells. The efficacy of this PTX/C6CP formulation was examined in vivo using cisplatin resistant xenograft A2780-CisR injected subcutaneously into the right flank and treated on days 0, 4, 8, and 12 via tail vein injection. The co-loaded nanoparticles significantly inhibited tumor growth and increased survival compared to the single drug loaded nanoparticles [76].

Using a similar prodrug approach, platinum prodrugs have been combined with doxorubicin using poly (ethylene glycol)-block-poly (L-lysine) based nanoparticles. Specifically, a Pt (IV) prodrug with aliphatic tail (C_16_) and carboxyl group as well as cis-aconitic anhydride doxorubicin were anchored to the amine groups of the lysine groups within the polymer forming polymer-prodrug conjugates. The resulting polymer-prodrug conjugates self-assembled into approximately 200 nm nanoparticles with both hydrophobic drugs in the core. The cytotoxicity of the nanoparticles was examined in vitro using A2780 and A2780DDP cells. The nanoparticle formulations increased potency of the platinum prodrug compared to the free prodrug as indicated by the decrease in IC50 value by 2.5 to 3.3-fold for A2780 and A2780DDP cells, respectively. This effect was drug dependent as formulation slightly decreased the potency of doxorubicin (increased IC50 in both cell types). The combination index was less than 1, indicating the drug combination was synergistic and more effective in the A2780DDP cell line. The dual drug loaded nanoparticle was a more potent than the free drug combination (lower combination index (*CI* 0.21 compared to 0.59). Further analysis indicated that the dual drug loaded nanoparticle was more effective at promoting cell apoptosis than the single free drug or single-drug loaded nanoparticles [77].

Cisplatin has also been combined with other classes of drugs. For example, cisplatin was formulated with wortmannin, a DNA repair inhibitor using PEG-b-PLA nanoparticles. Drugs containing nanoparticle cores were prepared using nanoprecipitation with PEG-b-PLA. The nanoparticle suspension was further stabilized by polyvinyl alcohol. The hydrophobic nanoparticle cores had a maximum cisplatin loading of 9.94 ± 2.07 wt.% and 1.59 ± 0.70 wt.% of wortmannin for single drug loaded nanoparticles. When co-loading the drugs, the presence of cisplatin increased the possible wortmannin loading to 2.89 ± 0.20 wt.%. Nanoparticle size was typically between 100 and 150 nm by dynamic light scattering. Evaluating the performance of the nanoparticle formulations in vitro, the cytotoxicity was measured using A2780 ovarian cancer cells. Formulating the drug into nanoparticles increased their toxicity by two-to four-fold compared to the free drug (based on decreases in IC50). The nanoparticles co-loaded with both drugs at a ratio of 1:1.4 wortmannin: cisplatin had a significant effect; a 21-fold decrease in IC50 of cisplatin was observed. The nanoparticle efficacy was measured in vivo using an A2780cis xenograft model by injecting cells into the right flank and treating the mice by tail vein injection followed by radiation. Treatment with the nanoparticles resulted in significantly reduced tumor growth rates compared to the free drugs. Additionally, increased cisplatin localization in the tumor was observed via immunofluorescence when treating with the nanoparticle formulations than the free drugs [78].

Cisplatin has also been combined with antioxidants such as (-)-Epigallocatechin-3-O-gallate (EGCG) in nanoparticles to reduce toxicity and increase efficacy. EGCG is the most abundant catechin in green tea and been shown to inhibit tumor growth. To incorporate ECGC into nanoparticles, it was conjugated to thiol-modified-hyaluronic acid. The resulting polymer was complexed with cisplatin. Upon self-assembly, cisplatin and EGCG were enriched in the nanoparticle core with a hyaluronic acid shell. This process was driven by hydrophobic interactions of the EGCG moieties. The resulting nanoparticle size was 109 ± 30 nm in water. The nanoparticles were used to treat SKOV-3 ovarian cancer cells in vitro. Intracellular uptake was possible facilitating Pt accumulation. Antitumor activity was examined in vivo using a subcutaneous SKOV-3 xenograft treated intravenously once a week for three weeks. Formulating the cisplatin with ECGC increased the Pt accumulation in the tumor and reduced tumor volume as well as increased survival rate compared to free cisplatin in vivo [79].

Similarly, platinum drugs have been combined with curcumin, a natural anticancer agent. For example, polymer micelle carriers encapsulating oxoplatin combined with curcumin, a natural antioxidant with anti-cancer activity, have also been studied. A triblock copolymer micelle was used to create a compartment for curcumin and one for conjugation of platinum drugs. Specifically, amphiphilic polycaprolactone (PCL)-based block copolymers were synthesized using ring opening polymerization and reversible addition fragmentation chain transfer (RAFT) polymerization. The resulting micelle had a PCL shell containing curcumin and a polyoligo (ethylene glycol) methyl ether methacrylate (POEGMA) shell (hydrophilic). A third, amine-containing monomer was added for conjugation to platinum. The chain was extended with 3-((tert-butocabonyl)amino)propyl acrylate (ABPA) to produce PCL-b-ABPA-b-POEGMA. Drugs were loaded in the nanoparticle following polymer self-assembly. Specifically, curcumin was loaded in the nanoparticle core while the Pt (IV) complex was used to crosslink the micelle. The resulting drug loaded nanoparticles were approximately 30 nm in diameter by dynamic light scattering. The cytotoxicity was measured using A2780 ovarian cancer cells. Formulating the drugs into micelles enhanced drug potency in vitro as indicated by the decrease in IC50 value. For example, the IC50 value in curcumin decreased three-fold when using the micelle formulations. Similar results were observed with platinum loaded drug micelles. Examining the drug combination, a strong synergistic interaction (CI~0.3) was observed when the drugs were co-loaded in the nanoparticles in comparison to a weakly synergistic interaction observed with free drugs (*CI* ~ 0.8). Interestingly, a weak synergistic interaction was also observed with co-delivery of two single-drug micelles. Thus, co-formulation of the two drugs appears to enhance treatment efficacy [80].

Polymer micelles containing triple drug combinations have also been accomplished. Liao et al. used ring-opening metathesis polymerization to prepare brush star polymers from macromonomers containing 3 kDa PEG and camptothecin or doxorubicin. Cisplatin was incorporated by designing a bis-norborene complex crosslinker containing Pt (IV). Upon reduction of the labile Pt-O bonds, cisplatin would be released. The nanoparticle size was affected by the macromonomer to crosslinker ratio. The resulting three drug loaded nanoparticles were 122 to 191 nm in diameter by dynamic light scattering. Their efficacy was evaluated in vitro using OVCAR-3 (platinum refractory human ovarian cancer cell line). Exposing the cells to 365 nm UV light for 10 min had no cytotoxic effects. In the presence of the nanoparticles, UV light triggered the release of doxorubicin leading to a 2.3-fold decrease in IC50 value (increased drug potency). The three drug loaded nanoparticle was more potent than the one-and two-drug loaded nanoparticles as indicated by the up to 11-fold decrease in IC50 (without or without UV light) [81].

Overall, encapsulating platinum drugs into nanoparticles in combination with other anticancer agents improves efficacy in vitro and in vivo due to synergistic drug interactions, improved pharmacokinetics due to controlled drug release, reduced toxicity to healthy tissues, and addition of targeting moieties to improve the spatiotemporal control of the two agents. Details of polymer nanoparticle formulation and the respective in vitro and in vivo results for micelles loaded with platinum and taxane drugs can be found in Table 1.

**Table 1:** Polymer nanocarriers coencapsulating platinum-based agents with other anticancer drugs.

Nanoparticle	Drugs	In Vitro	Key Results In Vitro	In Vivo	Key Results In Vivo	Source
Folic acid (FA)-PEGylated calix[4]arene nanoparticle	carboplatin/paclitaxel	SKOV-3, HO-8910	Encapsulation increased the cell mortality rate of SKOV-3 by 2.5-fold; conjugation further increased the cell mortality rate by 3-fold in vitro	SKOV-3 xenograft (armpit) treated once every other day via intratumor injection	Reduced tumor volume compared to the free drug	[73]
Folic acid (FA)-PEGylated-polypeptide-nanogels	cisplatin/paclitaxel	A2780/Luc	2-fold decrease in IC50 after 24 h using FA	A2780/Luc xenograft (IP) treated once every 4 days via tail vein injection	Increased cisplatin accumulation in tumor tissue; improved tumor inhibition and survival compared to single drug formulations	[75]
Poly(2-oxazoline) micelles	cisplatin/paclitaxel	A2780 and A2780cis (platinum resistant)	40:3 ratios of PTX to C6CP resulted in combination indexes less than 0.2 in A2780CisR cells; *CI* highly dependent on the drug ratio	A2780/Luc xenograft (right flank) treated once every 4 days via tail vein injection	reduced tumor growth, increased survival compared to single drug loaded micelles	[76]
PEG-poly-(L-lysine)	Cisplatin/doxorubicin	A2780/A2780DDP (platinum resistant)	2.5-to 3.3-fold decrease in IC50 of cisplatin, *CI* 0.21–55	-	-	[77]
PLGA-PEG	cisplatin/paclitaxel	SKOV-3	The co-loaded formulation was significantly more potent than prodrug stabilizer (3600-fold decrease in IC50)	-	-	[74]
PLGA-PEG NPs	cisplatin/wortmannin (DNA repair inhibitor)	A2780 and A2780cis (platinum resistant)	synergistically enhanced efficacy of A2780cis (*CI* ~ 0.04) with a 21-fold decrease in IC50, but were additive in A2780 cells (*CI* ~ 0.9–1.2)	A2780 and A2780cis xenograft (right flank) treated once by trail vein injection	reduced tumor growth rates compared to the free drugs; Increased cisplatin localization in the tumor	[78]
Hyaluronic acid micelles	cisplatin/EGCG	SKOV-3	Slight decrease in cell viability compared to single drug loaded NPs. Intracellular uptake was possible facilitating Pt accumulation.	SKOV-3-Luc xenograft (IP) treated once a week for 3 weeks by IP injection	increased the Pt accumulation in the tumor and reduced tumor volume as well as increased survival rate compared to free cisplatin	[79]
PCL-based triblock co-polymer micelle carriers	oxoplatin/curcumin	A2780	strong synergistic interaction (*CI* ~ 0.3)	-	-	[80]
poly(norbornene)-co-poly(ethylene glycol)	Cisplatin/doxorubicin camptothecin	OVCAR-3	The triple drug co-loaded formulation was more potent than the single drug (cisplatin) or two drug loaded combination as indicated by the decrease in IC50 (up to 11-fold)	-	-	[81]

### 3.3. Taxane Based Combinations

Polymer nanoparticle formulations of taxane-based drug combinations have also been considered (and summarized in Table 2). For example, Boztas et al. utilized poly (β-cyclodextrin triazine) (PCDT) to formulate combinations of paclitaxel and curcumin. Curcumin, a low-molecular weight polyphenol extracted from turmeric, has had synergistic effects when combined with paclitaxel using polymer and polymer nanoparticle platforms. Curcumin can inhibit the proliferation and survival or tumor cells as well as improve the drug resistance of tumor cells. Therapeutic applications of curcumin in the preclinical and clinical stages have been limited due to its low water solubility, low dissolution rate, and poor viability. Thus, formulation using polymers has been considered [82,83,84]. PCDT was synthesized through a one-step condensation polymerization of β-cyclodextrin triazine. The resulting polymer had a molecular weight of 25.7 kg/mol by GPC. Curcumin was incorporated by forming an inclusion complex with the PCDT in a mixture of acetone and water and freeze-drying. Then, the paclitaxel was complexed in a mixture of ethanol and water followed by freeze drying. The potency of the drug formulations were evaluated in vitro using various cancer cell lines including ovarian cancer cell lines A2780 and SKOV-3. The drugs were significantly more potent when complexed with the polymer. For example, there was a 15-fold decease in IC50 concentration in A2780 cells when compared to the free drug combination. There was also a five-fold decrease in IC50 concentration in SKOV-3 cells compared to the free drug combination. Interestingly, the drugs acted synergistically in the polymer complex. The combination index was 0.69 and 0.65 for A2780 and SKOV-3 cells, respectively. The combination index was lower than the free drug combinations in both cell lines (0.82 and 1.0 for A2780 and SKOV-3 cell lines, respectively). Quantitative apoptotic activity analysis by flow cytometry indicated that the polymer/drug combination increases apoptosis in A2780 and SKOV-3 cells compared to the free drug combination. The increase in drug efficacy suggest that the polymer platform improves drug solubility and bioavailability of the drugs [85].

Additional polymer-based nanoparticle formulations combining curcumin and paclitaxel have also been reported. Specifically, the drug combination was incorporated into polyethylenimine-g-stearic acid micelles coated with hyaluronic acid. The drug-loaded micelles were prepared by probe ultrasonication. Briefly, the drugs dissolved in ethanol were added to the micelles dispersed in water and sonicated, stirred, dialyzed, and centrifuged to remove unincorporated drugs, filtered (0.45 μm), and lyophilized. The drug loaded particles had a diameter of approximately 190 nm with a polydispersity index of 0.252 measured by dynamic light scattering. The potency of the nanoparticles was assessed in vitro using SKOV-3 and SKOV3-TR30 cells. Formulating paclitaxel into nanoparticles significantly increased its potency. There was a 12-fold decrease in IC50 for paclitaxel in SKOV-3 cells compared to the free drug. Interestingly, there was a larger effect of SKOV3-TR30 cells; a 72-fold decrease in IC50 for paclitaxel was observed compared to the free drug. Formulating curcumin with the polymer micelles also enhanced its potency. There was a 3.7-to 5.5-fold decrease curcumin IC50 upon formulation compared to the free drug for SKOV-3 and SKOV3-TR30 cells, respectively. Co-encapsulation of the drugs further increased paclitaxel potency. The IC50 was 17.3-fold lower in SKOV-3 cells and 115-fold lower in SKOV-3-TR30 cells compared to free paclitaxel. Notably, the co-loaded formulation was more potent than treating with free paclitaxel combined with curcumin nanoparticles. The formulation of the combination was also beneficial compared to the paclitaxel only nanoparticles; there was a ~1.5-fold decrease in IC50 of paclitaxel when formulating the combination compared to paclitaxel only. The in vivo efficacy was evaluated using a SKOV-3 xenograft model injected subcutaneously in the right side. Treatments were administered via tail vein injection every other day for 5 cycles. The co-loaded formulation reduced tumor volume in vivo compared to the free drugs. These results demonstrate paclitaxel and curcumin can be incorporated into polymer micelles with promising effects in vitro and in vivo [86].

In another approach, Devalpally et al. incorporated paclitaxel or tamoxifen in PCL nanoparticles stabilized by Pluronic F-108 (triblock copolymer of poly(ethylene glycol)-block-poly(propylene glycol)-block-poly(ethylene glycol)) via nanoprecipitation (with nanoparticle recovery via lyophilization). The resulting nanoparticles were spherical with a smooth surface and mean diameter of 200 nm. The efficacy of the nanoparticles was evaluated in vitro using SKOV-3 (drug sensitive) and SKOV-3TR (drug resistant) cell lines. Nanoparticle formulations of paclitaxel were significantly more potent than free paclitaxel as indicated by the 100-fold decrease in the IC50 value. Nanoparticle formulation of tamoxifen also improved the potency. There was a 10-fold reduction in IC50 for SKOV-3 cells and two-fold reduction in IC50 for SKOV-3TR cells. Xenografts of both cell types were implanted on the flanks of the mic. Treatments (paclitaxel, tamoxifen, or the combination) were administered via tail vein injection on day 1 and day 24. The free drug combination delayed tumor growth and increased the tumor volume doubling time. Furthermore, the drug combination of the nanoparticles delayed tumor growth and increased the tumor volume doubling time compared to the free drug combination in both SKOV-3 and SKOV-3TR xenograft models. Nanoparticle formulation also improved the safety profile of paclitaxel. Upon administration of the nanoparticles, there was no evidence of a significant increase in white blood cell count, acute liver toxicity or body weight compared to the control. In contrast, administration of the same dose of free drug resulted in noticeable adverse effects e.g., ataxia, decreased activity, and enhanced respiration. Overall, these results demonstrate administration of nanoparticle formulations is a promising approach in enhancing the cytotoxicity and reducing side effects of paclitaxel [87].

Another drug that has been formulated with paclitaxel is Tacrolimus. Tacrolimus is an effective inhibitor of P-glycoprotein (and ABC transporter) and may have an effect on overcoming drug resistance. Side effects have limited its clinical applications. Thus, formulation in combination with paclitaxel to reduce side effects and overcome drug resistance has been examined. Paclitaxel and tacrolimus were co-loaded in micelles of diblock copolymers composed of poly(ethylene glycol) (PEG) and poly (ε-caprolactone) (PCL) (PEG-b-PCL). The drugs and nanoparticles were dissolved in acetone and the solvent was evaporated to form a thin film. The thin film was dispersed in water at 60 °C to self-assemble the micelles. The resulting dispersion was filtered (0.22 μm) to remove any drugs that were not incorporated. The drug ratio was held constant by mass and the ratio of drug to polymer was increased. The drug loading increased as the nominal drug concentration increased; the encapsulation efficiency decreased. The maximum drug loading achieved was 38.5% with a hydrodynamic size of 36.4 ± 0.5 nm and polydispersity index of 0.09 ± 0.04 by dynamic light scattering. The potency of the combination formulations was assessed in vitro using A2780/T cells. The effect of paclitaxel to tacrolimus ratio was examined. The most potent ratio was 2 μg/mL paclitaxel to 16 μg/mL tacrolimus. The presence of tacrolimus in that ratio enhanced the paclitaxel potency; there was a 5.3-fold decrease in IC50 concentration compared to the paclitaxel-only micelle. Additionally, there was increased intracellular accumulation of paclitaxel in the when treating with the combination compared to the paclitaxel only micelle in A2780/T cells (a paclitaxel resistant cell line). There was also increased apoptosis and G_2_/M arrest by flow cytometry in cells treated with micelles containing the combination compared to paclitaxel-only. Taken together, these results indicated that the co-delivery system can sensitize these ovarian cancer cells in vitro to paclitaxel [88].

Paclitaxel has also been combined with tyrosine kinase inhibitors such as lapatinib to treat various types of cancer such as breast and prostate cancer. Some studies have formulated the combination for treating ovarian cancer cells. For example, Vergara et al. encapsulated lapatinib and paclitaxel in chitosan/alginic acid nanocapsules using layer-by-layer self-assembly. Briefly, chitosan, ammonium bicarbonate and drugs were sonicated in water to from paclitaxel/chitosan nanocores. Next, alginic acid layer solution was added and sonicated. Chitosan and alginic acid solutions were added sequentially to produce a three-bilayer capsule wall. The capsules were recovered by centrifugation. The in vitro toxicity of the capsules was evaluated using OVCAR-3 cells. The cytotoxicity was independent of drug concentration above 10 ng/mL (up to 20 μg/mL). The OVCAR-3 cells were considered resistant to paclitaxel and the IC50 concentration was not determined. Formulating the paclitaxel into nanocapsules improved the cytotoxicity compared to the free drug. Additionally, formulating the lapatinib/paclitaxel combination into nanocapsules resulted in higher cytotoxicity compared to the free paclitaxel [89].

Levit et al. incorporated paclitaxel and lapatinib into polymer nanoparticles using Flash NanoPrecipitation. Briefly, the drugs, tannic acid, and polystyrene-b-PEG stabilizer was dissolved in tetrahydrofuran and rapidly mixed with an aqueous FeCl_3_ solution using a confined impinging jet mixer. The effluent of the mixer was diluted immediately with PBS at pH 7.4. Upon mixing the tannic, acid-iron formed an insoluble complex that precipitated with the drugs to form the nanoparticle core. The core was stabilized in the aqueous dispersion by self-assembly of the amphiphilic block copolymer. The resulting nanoparticles were 100–200 nm in diameter by dynamic light scattering. The drug loading of the co-loaded particles was 2.1% paclitaxel and 0.8% lapatinib. The potency of the nanoparticle formulations was evaluated in vitro using OVCA-432 cells. Formulating the drugs into nanoparticles significantly increased their potency. For example, the IC50 of lapatinib decreased by approximately six-fold upon encapsulation. Interestingly, the IC50 of paclitaxel decreased by over 1500-fold from 70.6 ± 5.1 μg/mL to 0.040 ± 0.003 μg/mL. The significant increase in potency was attributed, in part, to sustained release over the 48-hour treatment period and the increased bioavailability of the formulation compared to the free drug. Combining lapatinib and paclitaxel further increased paclitaxel potency; there was a two-fold decrease in IC50. The combination index was 0.23, which was lower than co-delivered single drug nanoparticles (*CI* = 0.40). These results indicate that there is an advantage to incorporating both drugs into the same particle [90].

Another class of drugs that have been combined with paclitaxel are drugs that affect the metabolic activity of cancer cells. Cancer cells often rely on aerobic glycolysis for energy acquisition (the Warburg effect). Enzymes such as hexokinase 2 catalyze the first step of glycolysis and are overexpressed in many types of cancer. Drugs such as lonidamine inhibit hexokinase 2. Milane et al. developed nanocarriers for combination paclitaxel/lonidamine delivery. Specifically, the drug combination was incorporated into PCL nanoparticles and stabilized with EGFR targeting peptide grafted to PLGA-b-PEG and PEO (Pluoronic F-108 NF) via nanoprecipitation. The molar ratio of lonidamine to paclitaxel was 10:1 with a hydrodynamic size of 123.4 ± 4.4 nm. No burst release of lonidamine or paclitaxel was observed at pH 7.4 or 6.5; rather sustained release over several days. The efficacy of the nanoparticles was tested in vitro using various ovarian cancer cell lines including SKOV-3 and OVCAR-5 under normoxic and hypoxic conditions as well as a drug resistant SKOV3-TR cell line. Nanoparticles with the EGFR-peptide showed increased cell uptake in SKOV-3 and SKOV-3TR cell lines at early time points. Comparing the potency of the nanoparticle formulation to the free drugs in solution in terms of IC50 value, the results were cell type dependent. For the SKOV-3 cell line, formulation of the drugs did not significantly affect the potency of the drugs under normoxic or hypoxic conditions. In SKOV-3TR cells, there was also no significant effect on drug potency when comparing nanoparticle formulations to free drugs in solution. Interestingly, in OVCAR-5 cells, under normoxic conditions, the nanoparticles and free drugs in solutions had comparable IC50 values. Under hypoxic conditions, formulation the drugs into nanoparticles significantly increased the drug potency as indicated by the over two-fold decrease in IC50 value. Further, nanoparticles that facilitated the co-delivery of lonidamine with paclitaxel resulted in greater reduction in cell viability of MDR ovarian cells (SKOV-3TR, SKOV-3, OVCAR-5, OVCAR-5HYP) compared to the equivalent drug concentrations in solution as well as paclitaxel only nanoparticles. The results from the study suggest that lonidamine which affects cell glycolysis in combination with paclitaxel can have some benefit in combination with hypoxia. Further, this study demonstrates the potency of a nanoparticle formulation can be cell type dependent [91].

Formulation of triple drug combinations using paclitaxel have also been considered. Cho et al. combined paclitaxel with cyclopamine and gossypol using PEG-b-PCL micelles. Cyclopamine affects the hedgehog pathway activated in several cancer types as well as cancer cell types. Inhibiting of this signaling pathway can reduce growth of spheroid-forming ovarian cancer cell types (e.g., ES-2, SKOV-3, TOV-112D) as well as reverse taxane resistance. Gossypol inhibits anti-apoptotic proteins, e.g., Bcl-2, which is overexpressed and linked to cisplatin resistance in ovarian cancer. These three drugs are poorly water soluble. Drug loaded micelles were prepared by solvent evaporation i.e., nanoprecipitation. Specifically, the drugs and block copolymer were dissolved in acetone and added to saline or PBS at 60 °C with vigorous mixing. The acetone was evaporated under reduced pressure at 60 °C. Unincorporated, insoluble drugs were removed by centrifugation. The resulting dispersion was filtered (0.22 μm nylon). The resulting drug loaded micelles had a z-average diameter of 80–90 nm by dynamic light scattering and equal mass loading (6 mg/mL) of the three drugs. Interestingly, the three-drug micelles did not exhibit significantly more potency compared to paclitaxel loaded micelles in a 2D cell model (SKOV-3 and ES-2) based on IC50 values. However, in a 3D tumor spheroid model using ES-2-luc cells, upon treatment with 1 μM, the micelles with the drug combination resulted in disaggregation of the spheroid without a defined morphology. In contrast, spheroids treated with single drug and two-drug treatments retained their spherical morphology. The efficacy of the micelle formulation was assessed in vivo using ES-2-luc and SKOV-3-luc xenograft models. Treatment was administered via an intraperitoneal (IP) route once every seven days for three weeks. The three-drug micelles significantly reduced delayed tumor progression, reduced tumor volume and prolonged survival compared to paclitaxel-only micelles [92]. These results demonstrate the unique potential of formulations of drug combinations as well as the need to develop a better understanding of in vitro potency and in vivo efficacy.

Overall, taxane agents have been successfully paired with a wide range of anticancer drugs and antioxidants in micelles with enhance anticancer activity and synergy due the ability to overcome drug resistance. Details of micelles loaded with taxane in combination with other anti-cancer drugs can be found in Table 2.

### 3.4. Other Drug Combinations

While paclitaxel and platinum chemotherapeutic agents are the most prevalent in both free drug and nanoparticle formulations, nanoparticle formulations of other drug combinations have been investigated in ovarian cancer in vitro and in vivo and are summarized in Table 3. For example, Li et al. incorporated in docetaxel and gemcitabine on folate-targeted nanoparticles to treat ovarian cancer. Specifically, docetaxel and gemcitabine were co-loaded in nanoparticles stabilized by folic acid-conjugated to PEG-b-PLGA using the nanoprecipitation method. The drugs and modified polymer were dissolved in chloroform and then the chloroform was evaporated to form a film. The film was redispersed in PBS and homogenized for 10 cycles and 25,000 psi. The resulting dispersions were centrifuged to recover the free drug. Upon redispersion, spherical, drug-loaded nanoparticle formed by self-assembly. The resulting nanoparticle size was ~120 nm by TEM. The particle dispersions appeared to be stable over 3 months; no particle aggregation or change in particle size was observed. The cytotoxic effect of the nanoparticles was evaluated in vitro using SKOV-3 cells. Formulating the drug combination into nanoparticles increased the potency by over 3.5-fold as indicated by the decrease in IC50 value. The efficacy of the nanoparticles was studied in vivo using a xenograft model. The mice were treated via tail vein injection every 2 days for 3 weeks. At the end of treatment, the nanoparticles had significantly reduced tumor volume compared to the free drugs. Overall, the results showed the nanoparticles significantly reduced tumor volume, rate of tumor growth, and reduced off-target toxicity compared to free drug combination [93].

Doxorubicin is another widely used chemotherapeutic agent that has been used to treat numerous cancers including ovarian cancer. Thus, some nanoparticle formulations have studied drug combinations of doxorubicin. For example, doxorubicin and gemcitabine have been combined in micelles of diblock copolymers of polylactic acid and polyethylene glycol (PLA-b-PEG) based micelles. mPG-PLA-OH was conjugated to either gemcitabine or doxorubicin. Mixed micelles were prepared in aqueous solution by co-assembly of the two modified block copolymers due their amphiphilic nature. The resulting co-loaded micelles were 175–200 nm in diameter by dynamic light scattering. When evaluated in vitro, the drug loaded micelles appeared to internalize in SKOV-3 cells via endocytosis. Further, when formulated into micelles the drugs demonstrated enhanced potency (decreased IC50) and synergistic effects (*CI* < 1) in various cancer cell lines in vitro (liver and breast) [94].

Similarly, doxorubicin has been combined with verapamil, a calcium-channel blocker, to inhibit P-glycoprotein, a multi-drug resistance protein (ABC transporter). The two drugs were co-encapsulated by micellizing mPEG-PLA in the presence of the drugs by rehydration of thin films. The nanoparticle size was 25 nm and the drug loading of doxorubicin was 3% for doxorubicin and 0.6% for verapamil. Formulating the drugs in micelles increased drug accumulation and enhanced apoptosis in vitro using drug resistant ovarian cancer cell lines (A2780 and SKOV-3). The micelle formulations were tested in vivo using xenografts of the same cells in nude mice. Treatments were given every 3 days for two weeks via tail vein injection. The co-loaded micelles inhibited tumor growth and increased survival time compared to free doxorubicin with reduced side effects [95].

Doxorubicin has also been combined with irinotecan using micelles made from an amphiphilic polymer methoxy poly(ethylene glycol)-block-poly-(N-2hydroxethyl)-aspartamide modified with phenylboronic acid (mPEG-b-PHEA/PBA (PPBA). The phenylboronic acid moieties on the polymer side chains of the hydrophobic serve as electron acceptors to form coordination interactions with the drugs (electron donor containing). Drug loaded micelles were prepared by nanoprecipitation. Drugs were dissolved in DMF and neutralized by trimethylamine; the polymer dissolved in DMF was added. The drug/polymer mixture was added to water dropwise under mixing. The resulting suspension as dialyzed to remove the solvent and any unencapsulated drug. The drug loaded micelles were recovered by freeze drying. The coordination interactions were efficient at encapsulating the drugs; up to 50% drug loading of doxorubicin was achieved (compared to 7% for polymers without phenylboronic acid moieties). Co-loaded micelles had a comparable total drug loading 24.1% doxorubicin and 23.4% irinotecan (50% total, 1:1 w:w ratio of doxorubicin: irinotecan) with a hydrodynamic diameter of 30–40 nm. Cell viability in vitro was evaluated in SKOV-3 cells. The IC50 values of the micelles were higher than the free drugs. In this case, formulation of the drugs decreased potency. Formulation of the co-loaded micelles had a synergistic effect compared to the single drug loaded micelles (*CI* = 0.3) [96].

Adriamycin, a hydrochloride salt formulation of doxorubicin, has also been used in combination with polymer micelles formulations of combinations polyphenols to decrease cardiotoxicity when treating ovarian cancer. Specifically, amphiphilic Pluronic F-127 was used to encapsulate curcumin with either resveratrol and quercetin or curcumin within ~25 nm micelles. Various ovarian cancer cell lines (ES2-Luc (transfected with luciferase), A2780, A2780ADR) were treated with Adriamycin in combination with the micelle formulations in vitro. Adriamycin in combination with resveratrol and quercetin improved potency of Adriamycin. For example, in A2780 cells, there was a 10-fold decrease in IC50 value when treating with micelle formulations of resveratrol and quercetin compared to with Adriamycin alone. Synergistic effects (*CI* < 1) were also observed in A2780 cells. In vivo assessment was performed using ES2-Luc xenograft model (Adriamycin sensitive) in athymic nude mice). The mice were treated once a week for 4 weeks via tail vein injection. The combination of Adriamycin of micelles with resveratrol and quercetin reduced tumor volume and reduced cardiotoxicity (as indicated by cardiac troponin I levels and left ventricular function) compared to treatment with Adriamycin alone. These results suggest that combinations of polyphenols with chemotherapeutic drugs can improve efficacy while lowering systemic toxicity [97].

**Figure 5:**
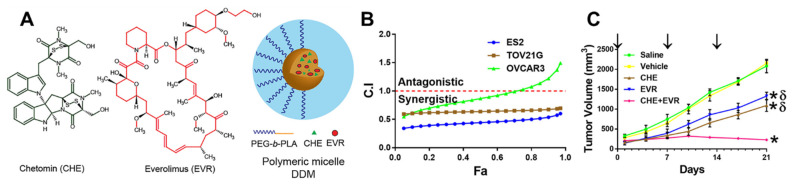
(**A**) Dual drug loaded polymer micelle loaded with chetomin (CHE) and everolimus (EVR) in the core. (**B**) The dual drug loaded micelles is synergistic compared to the single drug loaded micelles for TOV21G and ES2 cells in vitro (*CI* < 1). (**C**) The dual drug loaded micelles are more effective at reducing tumor volume compared to the single drug loaded micelles in an ES2 xenograft model. * Represents significant difference from control (saline), δ represents significant difference from dual drug loaded micelles evaluated using one way ANOVA with Tukey’s Multiple Comparison Test (compare all pairs of columns) using a significant level (α) of 0.05, n = 4. Adapted from [98]. Copyright 2019, with permission from Elsevier.

Using block copolymer micelles, combinations of chetomin and everolimus have also been examined (Figure 5A). Mutations in the tumor protein (TP52) and target of rapamycin (mTOR) pathway are considered key to invasive phenotype mutations in ovarian cancer under hypoxic conditions. Chetomin targets the hypoxic pathway; everolimus targets the mTOR pathway. Both are poorly water soluble. Thus, drugs were loaded into mPEG-b-PLA micelles using solvent evaporation. Single drug and co-loaded micelles were prepared by solubilizing the block copolymer and drug(s) in acetonitrile in a round bottom flask, evaporating the solvent, and rehydrating the film in deionized water at 60 °C. The micelles were recovered by centrifuging, and filtering. The resulting micelles were between 20 and 40 nm by dynamic light scattering. In vitro cell viability was measured in ES-2, OVCAR-3, and TOV-21G cell lines. Formulating the two drugs into the same micelle formulation had a synergistic effect as indicated by the *CI* < 1. The co-loaded formulation appeared to be most potent (lowest IC50 and CI) to ES-2 cells (Figure 5B). Thus, the in vivo efficacy was examined using and ES2 ovarian cancer xenograft model (injected subcutaneously into the right flank and treated on day 0, 7 and 14 via tail vein injection). The co-loaded micelles significantly reduced tumor volume compared to the single drug loaded micelles, empty micelles, and saline control (Figure 5C). No signs of toxicity were observed in vivo [98].

Another drug combination that has been studied in ovarian cancer treatment is floxuridine (water soluble antitumor drug) and chlorambucil (water soluble antitumor drug). Huang et al. used an amphiphilic drug-drug conjugate to form floxuridine-chlorambucil nanodrugs. Briefly, the hydroxyl of the floxuridine was conjugated to the carboxyl of chlorambucil by esterification with 1,3-dicyclo-hexylcarbodimide and 4-dimethylamino-pyridine. The resulting amphiphilic drug conjugate were self-assembled in water via nanoprecipitation from DMF followed by dialysis. The resulting size was 103 nm by dynamic light scattering with a polydispersity index of 0.143. TEM confirmed that the aggregates were spherical. The potency was evaluated in vitro using OVCAR-3 cells. The formulation of the nanoparticles from the drug conjugates were more potent that the free drug combination (lower cell viability and increases apoptosis). The combination index was also lower for the nanodrugs ~0.3 compared to ~0.7 for the free drug [99].

**Figure 6:**
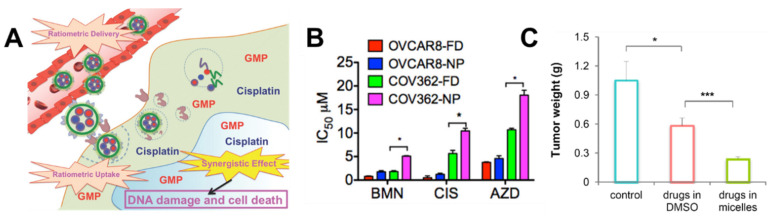
(**A**) Schematic overview of co-delivery of cisplatin and gemcitabine using polymer nanocarriers for synergistic effect (adapted with permission from [30] MDPI Open Access). (**B**) Co-formulation of cisplatin (CIS) and talazoparib (BMN) or olaparib (AZD) in lipid/polymeric nanoparticles (NPs) enhanced potency as indicated by the decrease in IC50 compared to the free drug (FD). * indicate a statistically significant difference between the IC50 of the free drug and nanoparticle formulation. Statistical analysis was performed by one-way ANOVA (*p* < 0.05). Reprinted (adapted) from [101] John Wiley & Sons Open Access. (**C**) Paclitaxel and tanespimycin (17-AAG) co-delivered in micelles (green) displayed the greatest suppression in tumor growth as indicated by the statistically significant reduced tumor weight on day 43 compared to the free drug combination (*, *p* < 0.05; ***, *p* < 0.0005, n = 6 mice). Reprinted (adapted) with permission from [112] PLoS Open Access.

Overall polymer micelle and nanoparticle-based systems have been a versatile platform for formulation of drug combinations to treat ovarian cancer (Figure 6A). These formulations have shown promising results in vitro and in vivo. Details of other drug combinations delivered in polymer micelles and nanoparticles are provided in Table 3.

### 3.5. Lipid Nanoparticles

Microemulsions and lipid based nanoparticles are a well-established platform for cancer treatment with improved safety compared to the free drug [18]. When used in combination with carboplatin and bevacizumab, PEGylated-liposomal doxorubicin has recently been proposed as the new standard clinical treatment for platinum-eligible recurrent ovarian cancer [100]. Adding polymers to the liposome formulations has also been used to enhance the structural stability and tune the drug release mechanism of drug combinations. For example, poly-L-lysine, a polycation, and hyaluronic acid, a polyanion were sequentially deposited on liposomes containing cisplatin (in the core) and either olaparib or talazoparib, poly(ADP-ribose (PARP) inhibitors (in the lipid bilayer). The resulting hybrid polymer-lipid nanoparticles were 90 ± 12 nm by dynamic light scattering. Due to the drug distribution in the nanoparticle structure, sequential release of the PARP inhibitors followed by release of the cisplatin was achieved. Examining the drug potency in vitro, formulating the drugs enhanced potency compared each of the free drug in both OVCAR-8 and COV-362 cells as indicated by the decrease in IC50 value (Figure 6B). The in vivo efficacy of the nanoparticle formulations was examined using mice with OVCAR-8 xenografts intraperitoneally injected and treated by tail vein injection every week. The nanoparticle formulation of the drug combination was a more effective treatment than the free drug combination at reducing tumor burden and metastasis as well as increasing survival [101].

Solid lipid nanoparticles are also a promising platform to achieve controlled drug release because the drug mobility in a solid lipid is expected to be significantly lower than a lipid in the liquid phase. Solid lipid particles can be prepared by freeze spray drying and used for retarded release after peroral administration. Aqueous dispersions of submicron particles are typically produced via high pressure homogenization of lipid, water, and an emulsifier (which has been found to be more effective than high shear mixing or ultrasound). Dilution of microemulsions has also been used to achieve dispersions of solid lipid nanoparticles with average particle size below 500 nm. Typically, the dispersions are stabilized with emulsifiers such as triglycerides, fatty acids, or steroids. Amphiphilic block copolymers such as Poloxamers (Pluronics, triblock copolymers of polyethylene glycol-b-polyoxypropylene-b-polyethylene glycol) have also been used as stabilizers. Lipid nanoparticles can encapsulate hydrophobic drugs in the core by dispersing the drug in the lipid prior to homogenization. Alternatively, drugs can be conjugated to the lipid. Detailed reviews on the formulation of solid lipid nanoparticles can be found elsewhere [102,103]. Lipid based nanoparticles can be combined with polymers to achieve controlled release drug (small molecule) cocktails. In this review, we highlight examples of lipid polymer hybrid nanoparticles used to deliver combinations of chemotherapeutics to treat ovarian cancer. Details of lipid-polymer nanocarrier studies are found in Table 4.

Using lipid based nanocarriers, Taxol has been combined with other drugs including curcumin and tetrandrine. For example, a combination of paclitaxel with curcumin (which downregulates ABC transporters) have been encapsulated PEG-modified nanoemulsions. Lecithin and a PEG-based lipid was added to water while paclitaxel in chloroform or curcumin in ethanol were added to flaxseed oil. The solvents were evaporated, then the nanoemulsions were prepared by coarse homogenization followed by high energy ultrasonication. The resulting hydrodynamic diameter was under 150 nm with a polydispersity index 0.3 or below. The cytotoxicity of the nanoemulsions of the single drugs and in combinations were measured on sensitive (SKOV-3) and resistant (SKOV-3TR) ovarian cancer cells. Formulation of the nanoemulsion increased the drug potency of paclitaxel by 1.8-fold in SKOV-3 cells (as indicated by the decrease in IC50 concentration compared to free paclitaxel). A similar effect was observed using SKOV-3TR cells. When used the nanoemulsions were used in combination, a slightly synergistic (*CI* = 0.93) in both drug-sensitive cells (SKOV-3) and additive in drug-resistant cells (SKOV-3TR) when compared to the free drugs was observed. Since the effect was not cell type dependent, curcumin was thought to inhibit the P-glycoprotein in both SKOV-3 and SKOV-3TR cell types [104]. Additional drug combinations have been examined. For example, tripterine and brucea oil, natural medicines for cancer treatment have been combined in microemulsions stabilized by PEG400 combined with other surfactants (e.g., Tween 20 or Tween 80). The cytotoxicity was evaluated in vitro using SKOV-3 cells. At an intermediate 20:1 w:w ratio of brucea oil to tripterine, there was a synergistic effect as indicated by the combination index of 0.90. At higher and lower ratios of brucea oil to tripterine the effect was antagonistic [105].

Similarly, Zhang et al. found used a lipid-polymer hybrid nanoparticle platform to co-deliver paclitaxel and tetrandrine (P-glycoprotein inhibitor). Paclitaxel was conjugated to PLGA and co-loaded into lipid polymer nanoparticles with tetrandrine via nanoprecipitation. The particles were stabilized by a combination of lecithin and PEG-based lipid (maleimide-PEG-DSPE). Finally, iRGD peptide was conjugated to the preformed nanoparticles (thiol-maleimide). The resulting particles were below 140 nm in diameter with polydispersity indexes 0.18 or below. The drug loading was approximately 10% and 7% for paclitaxel and tetrandrine, respectively. The cytotoxicity was evaluated in vitro using an ovarian cancer cell line (A2780). The co-delivered drugs showed synergy as indicated by the combination index between 0.49 and 0.65, depending on the drug ratio. They also observed increased the intracellular paclitaxel accumulation thereby increasing apoptosis in A2780/PTX cells which was mediated by tetrandrine [106]. Interestingly, the efficacy of this nanoparticle platform could, in part, be attributed to the core-shell structure which facilitated sequential release of tetrandrine prior to paclitaxel.

Lipid formulations are versatile and other drug combinations can be considered. A unique lipid formulation was created by Lee at al., in which cisplatin was co-delivered with doxorubicin lipid-based nanoparticle described as a single polymer-cage nanobin. Doxorubicin-loaded liposomes (with a mean hydrodynamic diameter of 98 ± 12 nm) were prepared and used as a lipid-based template for the nanobin. Cholesterol-terminated poly(acrylic acid) chains were then inserted into the lipid bilayer of the liposome and crosslinked to form the polymer cage of the nanobin. Finally, cisplatin prodrug, cis-[PtII(NH_3_)_2_(N_α_-AcLys)] was conjugated to unreacted carboxyl groups of the polymer cage. Following this step, the nanobin size was 128 ± 16 nm. The resulting platinum release kinetics were pH-tunable with increased release kinetics at lower pH due to the acid lability of the N_α_-acetylamido ligand. Effective release of doxorubicin at acidic pH was also observed. The Pt to doxorubicin ratio was systematically studied in an OVCAR-3 ovarian cancer cell line. Increasing the molar ratios of Pt to doxorubicin increased potency as indicated by the decrease in IC50 concentration of doxorubicin. Increasing the molar ratio of Pt to doxorubicin also increased synergistic effects as indicated by the decrease in combination index. Impressively, the combination index at a Pt to doxorubicin ratio of 6 in the nanoparticle platform was 0.27, which was significantly more synergistic than the free drugs used at the same ratio (*CI* = 0.90). The in vitro potency of this nanoparticle platform was attributed to enhanced cellular uptake and acid triggered drug release [107]. These results are consistent with other lipid polymer nanoparticles formulations of combinations of platinum-agents and Taxol studied for other forms of cancer (e.g., cervical) with synergistic drug interactions [108,109]. Other formulations of drug combinations encapsulated in lipid-polymer nanoparticles have displayed synergistic activity such as paclitaxel with doxorubicin [110] and doxorubicin with indocyanine green [111] in other forms of cancer.

Some lipid formulations of drug combinations have been evaluated in vivo using ovarian cancer cell lines. For example, paclitaxel was combined with tanespimycin (17-AAG), which is an inhibitor of Heat shock protein 90 (Hsp90), a highly conserved chaperon protein required for activation and stability of oncogenic kinases and transcription factors vital to tumor cell survival. Therapeutic use of 17-AAG has been limited by its poor water solubility. Further, combination therapy of 17-AAG with paclitaxel has been affected by dose-limited toxicities caused by the drugs and solvents used to solubilize the poorly water-soluble drugs. To overcome the toxicity of the excipients, formulation of the drug combination into nanoparticles has been reported. The drugs were co-loaded into mixed micelles of 1,2-Distearoyl-sn-glycero-3-phosphoethanolamine-Poly(ethylene glycol) (DSPE-PEG) and D-α-tocopheryl polyethylene glycol succinate (TPGS). Briefly, the drugs, PEG-DSPE, and TPGS were dissolved is chloroform. The chloroform was evaporated to form a thin drug-lipid film. The film was hydrated in buffer while vortexing. The resulting dispersion was centrifuged to remove any undissolved components. The resulting drug loaded nanoparticles had an average hydrodynamic diameter of 11 nm. The dispersion contained 1.45 mM paclitaxel and 4.0 mM 17-AAG by HPLC. The therapeutic potential of the micelle formulation was examined in SKOV-3 xenograft bearing mice. Specifically, the pharmacokinetics following a tail vein injection was compared to the free drugs dissolved in DMSO. At equal drug doses, the micellar formulation resulted in an over 10-fold increase in paclitaxel plasma concentration. The drug concentration in normal organs and elimination half-lives of the drug were not significantly affected by formulation into micelles. Labeling the nanoparticles with a near infrared dye and followed by whole body fluorescence imaging suggested that the micelles circulated intact. Notably, formulating the drugs into micelles also increased drug accumulation in the tumor. There was also a 3.5-fold increase in paclitaxel concentration and 1.7-fold increase in 17-AAG concentration in the tumor after a single injection of the micelle formulation compared to the free drug combination. Building on these results, tumor bearing mice received the drug combination on days 0, 7, and 14 as the free drug or micelle formulation. On days 3, 10, and 17, two groups were treated with 17-AAG as the free drug or micelle formulation. The treatment dose and schedule were selected based on what is done clinically. Formulating the drugs into micelles significantly reduced tumor volume (Figure 6C) and tumor weight on day 43 compared to the free drug [112].

Platinum agents have also been paired with various other anticancer agents such as gemcitabine to treat ovarian cancer using lipid-based formulations. For example, carboplatin prodrugs (carbo-bis (phosphonic acid)) was synthesized self-assembled with gemcitabine monophosphate in the presence of a Zn-based nanoscale coordination polymer via Zn-phosphate interactions. Dopamine was self-assembled onto the surface of the drug-containing core via hydrophobic interactions so that the particles could be dispersed in organic solvents. Finally, the nanoparticle was coated by a PEG-based lipid. The resulting diameter was 85 nm by dynamic light scattering. The carboplatin loaded was 28.0 ± 2.6 wt.% and the gemcitabine loading was 8.6 ± 1.5 wt.%. The particle size was stable in bovine serum albumin in phosphate buffered saline at 37 °C after 24 h. In vitro cytotoxicity was evaluated using SKOV-3 and A2780 ovarian cancer cells. Combining the drugs increased the potency as increased by the decrease in IC50 value. A synergistic drug interaction (combination index, *CI* ~0.5) was observed when these nanoparticles were used to treat platinum-resistant cells (A2780/CDPP) in vitro. Formulating the drug combination into nanoparticles increased cell apoptosis measured by flow cytometry. Evaluating the nanoparticle efficacy in vivo the antitumor activity was evaluated on SKOV-3 and A2780/CDDP subcutaneous xenograft murine models. Treatments were given once every three days via intraperitoneal injection. The nanoparticle formulations decreased the tumor weight by~90-fold and 12-fold for A2780 and SKOV-3 cells compared to the controls, respectively. Animals treated with the free drug combinations showed similar tumor growth as the control [113].

Similarly, curcumin has been combined with triptolide, a medicinal Chinese herb with antitumor potential in the treatment of ovarian cancer. Co-encapsulation of both fat-soluble compounds in mPEG-DPPE (dipalmitoyl-sn-glycero-3-phosphoethanolamine-N—[mPEG])/calcium phosphate in ~170 nm nanoparticles was achieved by emulsification). When evaluated in vitro using SKOV-3 cells, a higher apoptosis rate was observed with the nanoparticles compared to the free drugs after 24 h of treatment. When administered in vivo, reduced tumor growth with minimal off target toxicity was observed in mice with in SKOV-3 xenografts treated twice a week for 24 days compared to the free drug combination. These results were attributed to the presence of curcumin which reduction of ROS caused by triptolide [114].

Overall lipid-polymer based systems have been a versatile platform for formulation of drug combinations to treat ovarian cancer. Recent advances have shown promising results in vitro and in vivo. Details of lipid-polymer nanocarrier studies are summarized in Table 4.

**Table 4:** Lipid-polymer nanocarriers for co-delivery of anticancer drugs.

Nanoparticle	Drugs	In Vitro	Key Results In Vitro	In Vivo	Key Results In Vivo	Source
Polyelectrolyte coated liposome	Cisplatin/olaparib or talazoparib	OVCAR-8 and COV362	enhanced potency (reduced IC50) compared to free drugs	OVCAR-8 xenografts treated by tail vein injection once a week	Reduced tumor burden and metastasis as well as increasing survival	[101]
PEGylated lipid nanoemulsion	paclitaxel/curcumin	SKOV-3, SKOV-3TR (drug resistant)	enhanced cytotoxicity and increased apoptosis, slightly synergistic (*CI* = 0.93) in SKOV-3 and additive in SKOV-3_TR_ compared to free drugs	-	-	[104]
PEG stabilized microemulsion	brucea oil/tripterine	SKOV-3	*CI* = 0.90 at an 20:1 w:w ratio of brucea oil to tripterine	-	-	[105]
iRGD peptide Lipid-polymer hybrid nanoparticles	paclitaxel/tetrandrine	A2780/PTX cells (paclitaxel resistant)	*CI* 0.49–0.64 depending on drug ratios; increased intracellular paclitaxel accumulation apoptosis when co-loaded	-	-	[106]
Lipid-templated polymer-caged nanobins	cisplatin/doxorubicin	OVCAR-3	enhanced cytotoxicity compared to both free drug and single-drug nanobins; CI between 0.27 and 0.67 depending on drug ratio compared	-	-	[107]
DSPE-PEG micelles	paclitaxel/tanespimycin (17-AAG)	-	-	SKOV-3 xenograft (flank) with sequential delivery of paclitaxel (free or NPs) once a week and followed by 17-AAG (free or NPs) 3 days later for 3 weeks, administed through tail vein injection	increased tumor accumulation by 2-fold,~2-fold reduction in tumor mass after 43 days significant tumor growth arrest compared to free drug combinations	[112]
Core-shell DOPA, DSPE-PEG, and cholesterol nanoparticles	carboplatin/gemcitabine	SKOV-3, A2780/CDPP (platinum resistant cells)	*CI*~0.5 comparable to free drugs	SKOV-3, A2780/CDDP (platinum-resistant) xenografts (right flank) injected by IP once every 3 days for a total of 3 injections	reduced tumor weight by 12-fold compared to free drug combination	[113]
mPEG-DPPE/calcium phosphate nanoparticle	triptolide/curcumin	SKOV-3	Higher apoptosis rate compared to free drugs	SKOV-3 xenografts (upper left axillary fossa) treated twice a week for 24 days via tail vein injection	Reduced tumor volume compared to free drugs	[114]

### 3.6. Dendrimers

As an alternative to assembled polymer micelles and nanoparticles, another versatile polymer nanoparticle platform for drug delivery has been dendrimers. Dendrimers are hyperbranched, three-dimensional polymers. Starting from a core, they contain layers of branched repeating units and end groups on the outer layer of repeat units. Dendrimers are produced using iterative reactions. Each repeated reaction results in an additional layer of branches, i.e., a generation. The properties and resulting structure of the dendrimer can be tuned. For example, the polymer composition and number of branches will affect the size, hydrophobicity, surface charge. For drug delivery applications, therapeutic moieties can be covalently conjugated to polymer branches or entrapped in the dendrimer core by electrostatic or hydrophobic interaction. More detailed reviews of dendrimer synthesis and formulation can be found elsewhere [115,116,117,118]. In this review, we highlight examples of dendrimers used to deliver combinations of chemotherapeutics to treat ovarian cancer. Details of these dendrimer studies are found in Table 5.

**Figure 7:**
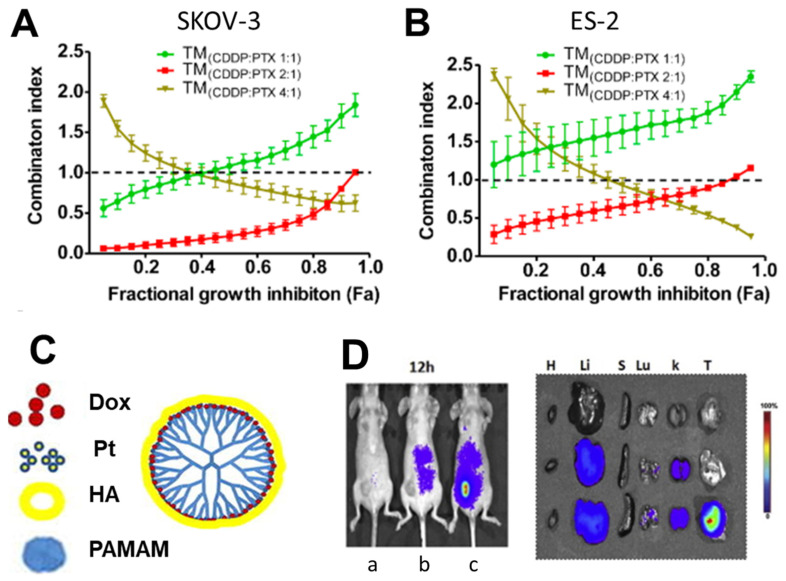
Combination index of (**A**) SKOV-3 and (**B**) ES-2 cells treated with telodendrimer nanocarriers loaded with paclitaxel and cisplatin (CDDP) at different ratio. The telodendrimers produce different drug interaction due to drug ratio and cell type. Reprinted from [119], Copyright 2015, with permission from Elsevier. (**C**) Schematic of polyamidoamine (PAMA) dendrimer formulated with hyaluronic acid (HA) loaded with cisplatin (Pt) and doxorubicin (Dox). (**D**) In vivo co-delivery of cisplatin with doxorubicin in polyamidoamine dendrimers enhance drug accumulation in tumor tissue facilitated by hyaluronic acid targeting ligands. (a: saline; b: Cy7.5-labeled PAMAM; c: Cy7.5-labeled HA@PAMAM). Reprinted from [120], Copyright 2019, with permission from Elsevier.

The bottom up synthesis of dendrimer provides a versatile platform for co-delivery of drug combinations. For example, three-layered linear-dendritic telodendrimers have been developed to co-deliver paclitaxel and cisplatin. PEG-based dendrimers were synthesized via a combination of solution phase peptide chemistry and “thio-ene” with MeO-PEG-NH_2_ and PEG-5K(COOH) conjugated to cholic acid (CA) using a triethylene glycol linker. The resulting telodendrimer was 9.0 ± 2.6 nm. The two drugs were encapsulated in a two-step method. Cisplatin was added to the dendrimers in water. Unbound drug was removed by ultrafiltration and the cisplatin-loaded telodendrimers were recovered by freeze drying. The recovered cisplatin-loaded telodendrimers were dissolved in chloroform with paclitaxel. The chloroform was evaporated and the telodendrimers co-loaded with cisplatin and paclitaxel were redispersed in water. Unbound paclitaxel was removed by filtration. The co-loaded nanoparticles increased in size to ~20 nm. Interestingly, drug release of paclitaxel was faster than cisplatin (50% release within 24 h compared to 50% drug release within 92 h, respectively). The drug ratio was varied, and the efficacy was compared in vitro using SKOV-3 and ES-2. Cai et al. found that at a ratio of 2:1 cisplatin to paclitaxel was highly synergistic for SKOV-3 (*CI* = 0.21). They were also weakly synergistic for ES-2 cells (*CI* = 0.65). This drug ratio was more effective than a 4:1 cisplatin:paclitaxel ratio. Notably, a 1:1 drug ratio was antagonistic (*CI* >1) in all cell lines. Building on the in vitro results, an in vivo studied was performed using a SKOV-3 xenograft model dosed at four-day intervals by tail vein injection. The dendrimer nanoparticle (2:1 cisplatin: paclitaxel drug loading) platform showed increased accumulation in the tumor site compared to free cisplatin. Further, the tumor volume was lower and the survival was longer when treating with the co-loaded dendrimers compared to free cisplatin, or single drug loaded telodendrimers (Figure 7A,B) [119]. Overall, encapsulation these drugs into dendrimers could lead to synergistic effects but were dependent on the drug ratio. Similarly, cisplatin and doxorubicin were co-delivered using polyamidoamine dendrimers. Paclitaxel and doxorubicin were conjugated to fourth generation polyamidoamine dendrimers (HA@PAMAM-PT-Dox). When conjugated to the dendrimers the drugs were approximately three times more potent than the free drug in a breast cancer cell line. Additionally, the dendrimer platform suppressed tumor growth compared to free drugs and single-drug loaded dendrimers in vivo (Figure 7C,D) [120].

Pathak et al. reported another approach for delivering drug combinations to treat cancers which involved incorporating drugs into the dendron backbone. Specifically, aspirin and cisplatin were incorporated at the termini of PLA dendrons. The resulting functionalized biodegradable polymer was formulated with PLGA-b-PEG into a nanoparticle cocktail. Examining drug release, Pt was released much faster than aspirin. The dendron based formulations were more potent than free Pt as indicated in the approximately four-fold decrease in IC50 value when evaluated in vitro in a cisplatin resistant ovarian cancer cell line (A2780) [121].

Alternatively, oxaliplatin has been used in combination with dendrimers containing curcumin. PEG dendrimers were synthesized from esterification of oleoyl chloride, polyethylene glycol in the presence of triethylamine. The resulting dendrimer and curcumin were dissolved in acetone and nanoprecipitated in PBS and filtered. Treatment efficacy of the curcumin loaded dendrimers with oxaliplatin was evaluated in vitro using SKOV-3 and OVCAR-3 cells. The combination had a synergistic effect. There was a 2.5-fold decrease in the IC50 concentration of oxaliplatin; the combination index (IC50) was 0.855 in SKOV-3 cells 48 h after treatment. The combination was more effective in OVCAR-3 cells as the *CI* was 0.708 [122].

Dendrimers combining paclitaxel and other anticancer agents have also demonstrated synergistic activity. For example, Zou et al. combined paclitaxel with borneol, a traditional Chinese medicine that inhibits P-glycoprotein in PEG-poly(amidoamine) dendrimers via a one-step precipitation. The resulting particles were approximately 90 nm in diameter. The potency of the formulations were tested in vitro A2780 cells and paclitaxel resistant A2780 cells. After 72 h of treatment, there was a 1.5-fold decrease in IC50 value compared to the free drug combination in the A2780 cell line. The increase in potency was more pronounced in the paclitaxel resistant cell line as the dendrimer formulation had a three-fold lower IC50 value compared to the free drug combination. The authors observed that the P-glycoprotein inhibiting activity of borneol increased the intracellular paclitaxel concentration. In vivo, treatment with the dendrimers (by tail vein injection once every two days for 14 days in BALB/c nude mice with A2780/PTX cell xenografts subcutaneously injected in the back) resulted in a significantly larger decrease in tumor volume compared to the free drug combination [123].

Leveraging their versatility, dendrimers have also been used for delivery of additional drug cocktails to treat ovarian cancer. For example, PEG-based telodendrimers have been used for co-delivery of bortezomib and doxorubicin. Bortezomib is a proteasome inhibitor which can sensitize cancer cells to DNA-damaging agents such as doxorubicin by inhibiting NF-κB activation (i.e., nuclear factor kappa-light-chain-enhancer of activated B cells). Bortezomib has a dipeptide structure and was conjugated to the intermediate layer of a three-layered telodendrimer. The interior layer was a doxorubicin binding layer. The resulting telodendrimers self-assembled with doxorubicin; the resulting hydrodynamic size was 20–40 nm. The efficacy of the telodendrimer platform was evaluated in vitro using SKOV-3 ovarian cancer cells. When formulated using the telodendrimer platform, the drug interaction was concentration dependent. At low bortezomib: doxorubicin ratios (e.g., 1:4), antagonism was observed. Synergistic effects were observed at bortezomib: doxorubicin ratios between 1:1 and 1:10. The efficacy of the telodendrimer platform was also evaluated in vivo in SKOV-3 ovarian cancer xenograft-bearing mice. The drug cocktail was dosed every 4 days via tail vein injection. After 3 doses, the mice treated with the nanoparticles showed delayed tumor growth compared to mice treated with free drug. The enhanced treatment effect in vivo was attributed to reduced systemic toxicity and synchronized drug availability at the tumor site [124].

In another example, Zhang et al. reported using azide-alkyne click chemistry to synthesize mPEGylated dendrimers containing peptide (GFLG) and doxorubicin. The peptide GLFG was attached to the periphery of the dendrimer to conjugate doxorubicin and used to achieve intra-lysosomal release of doxorubicin in the presence of Cathepsin B, a lysosomal cysteine protein that is overexpressed in many tumor cells. The resulting particle size was approximately 90 nm. In vitro, the IC50 of doxorubicin was 22-fold higher than free drug when measured using SKOV-3 ovarian cell line. In vivo, the efficacy of the dendrimer was evaluated in vivo in SKOV-3 ovarian cancer xenograft–bearing mice. Treatments were given via tail vein every 4 days for 17 days at a dose of 5 mg doxorubicin/kg. When treating with the nanoparticles, there was a significant decrease in tumor growth compared with free drug. Interestingly, in vivo performance was better than in vitro performance [125]. Similar approaches have been coordinated with multiple drugs for sequential drug release. For example, pH-responsive dendrimers (poly (propylene imine-based) have been used to deliver drug combinations of methotrexate and tretinoin, a vitamin A derivative. Slight improvement in drug efficacy were observed (e.g., decrease in IC50 value) [126]. Synergistic or enhanced anti-cancer activity has also been found with a variety of dendrimer drug combinations in lung and breast [127]. The work related to ovarian cancer is highlighted in Table 5.

**Table 5:** Dendrimer based nanocarriers for co-delivery of anticancer drugs.

Nanoparticle	Drugs	In Vitro	Key Results In Vitro	In Vivo	Key Results In Vivo	Source
PEG 3-generation telodendrimer micelles	cisplatin/paclitaxel	SKOV-3, ES-2	Antagonistic at 1:1 ratio; synergistic at 2:1 ratio of cisplatin to paclitaxel (*CI* = 0.21 for SKOV-3)	SKOV-3 xenograft (flank) treated 3 times at 4-day intervals via tail vein injection	highest accumulation in the tumor tissue, decreased tumor volume, increased survival time, and reduced renal toxicity compared to free cisplatin, cisplatin loaded telodendrimers, or paclitaxel loaded dendrimers	[119]
PLA/PLGA/PEG dendrimers	cisplatin prodrug/aspirin prodrug	A2780/CP70 (cisplatin resistant)	~4-fold decreased IC50 in nanoparticle formulation	-	-	[121]
PEG dendrimers	Oxaliplatin/curcumin dendrimers	SKOV-3/OVCAR-3	*CI* 0.855 in SKOV-3/*CI* 0.708 in OVCAR-3 after 48 h of treatment (IC50)	-	-	[122]
3-generation PEG-PAMAM dendrimers	paclitaxel/borneol	A2780/PTX (paclitaxel resistant)	3-fold lower IC50 value compared to the free drug combination	A2780/PTX xenograft (back) once every two days for 7 tail vein injections	decrease in tumor volume, compared with the free drug combination	[123]
Linear-dendritic telodendrimers	doxorubicin/bortezomib	SKOV-3	Synergistic effects observed at bortezomib: doxorubicin ratios between 1:1 and 1:10; antagonistic at lower ratios	SKOV-3 xenograft (flank) treated every 4 days for a total of 3 tail vein injections	decreased toxicity delayed tumor growth compared to free drugs	[124]

## 4. Outlook

Overall, polymer nanoparticles are a promising platform for co-delivery of drug combinations for ovarian cancer treatment. Advances in nanoparticle design can improve drug delivery by addressing drug resistance [4,28,30,31,69,71]. Specifically, sequential combination therapy with free drug formulations has been shown to improve drug efficacy and overcome resistance mechanisms. Therefore, the ability to co-encapsulate multiple therapeutics could be combined with the ability to control the sequence (i.e., temporal control) of release of the multiple therapeutics from the nanoparticle platform to expand on the potential of this technology. Sequential release of co-encapsulated drugs from a single nanoparticle would enable spatiotemporal release of the drug cocktail [28,30,31], which may further enhance treatment efficacy.

Achieving nanoparticles with tunable release of multiple drugs would be advantageous [28,30,31]. To tune the release rate of the drugs, various approaches have been used such as prodrug synthesis [128]. Alternatively, the different drugs can be loaded in different layers of the nanoparticles to achieve different release profiles [28,31,71]. Formulating stimuli-responsive nanoparticles is another approach for controlled release of drug combinations. Specifically, pH responsive nanoparticle platforms as well as enzyme responsive (e.g., protease, hydrolase, oxidase) have been used as a basis to achieve triggered release in biological environments. Full descriptions and reviews of stimuli-responsive nanoparticle platforms are outside the scope of this review can be found elsewhere [129,130,131,132]. Moving forward, stimuli-responsive platforms to control drug release may help address the gap between pre-clinical and clinical studies to improve treatment efficacy as well as patient compliance.

To date, few studies have examined sequential drug delivery with nanoparticles in ovarian cancer. For example, the effect of sequence of nanoparticle formulations of paclitaxel and lapatinib (a tyrosine kinase inhibitor) on treatment efficacy using OVCA-432 and OVCAR-3 was examined. The single drug loaded nanoparticles of each drug were prepared by Flash NanoPrecipitation (FNP) and delivered either simultaneously, paclitaxel followed by lapatinib, or lapatinib followed by paclitaxel. Overall, treatment with paclitaxel followed by lapatinib resulted in lower cell viability than the reverse order [133]. Similar results were observed with A2780 cells treated with paclitaxel and gemcitabine N-(2-hdyroxypropylmethacryamide) copolymers [134]. These results demonstrate that dosing schedule can be used to enhance efficacy of nanoparticle formulations and can be extended to other ovarian cancer cell types and facilitate design of nanoparticles carrying drug combinations. Tuning dosing schedules of drug combination has significant potential to improve treatment efficacy and overcome drug resistance mechanisms. Previous work has shown that the drug sequence, dose schedule, and drug ratio is cell-specific. However, screening various drug combinations and treatment schedules is time-and resource-intensive. Furthermore, conventional approaches of dose-response quantification do not provide a comprehensive understanding of complex biological systems and mechanisms of synergy.

Leveraging a systems biology modeling can provide a deeper understanding of the interaction of drug combinations when delivered from nanoparticles. This approach is based on drug activated signaling cascades and protein interaction networks. Several different methods have been developed to facilitate modeling of drug-cell interactions. Protein-protein interaction (PPI) and dynamic pathway simulation provide an understanding of which protein interactions induce synergism thereby providing the mechanism of action. The PPI network model is based on the functional associations of key proteins (activations/inhibition) and drug targets which provide information about feedback structure and cascade pathways of drug targets [135]. Dynamic pathway simulation models the dynamic behavior of drugs and mechanisms of action which is based on concentrations and activity levels targets which yields dose-response data [135]. The advantage of these two models is that they provide an understanding between the mechanism of action and synergy. However, they are either complex or not all encompassing. Instead, network motifs can be used to describe the same number of events in distinct patterns. This alternative method helps to distinguish which characteristic of the pathway produces synergistic activity. These three models can be used to determine synergistic drug combinations and minimal effective dosages [136]. The network approach has been used to model treatment of carboplatin-and paclitaxel-resistant ovarian cancer. The model found that delivering carboplatin followed by ABT-737 (a Bcl-2 inhibitor) produced the greatest synergism [136,137]. Other approaches include stochastic differential equations (SDEs) based model to address cell heterogeneity and adaptability. This method has been used to identify dose-dependent synergistic effects as well as patterns between drug resistance mechanisms and population-level patient survival [138]. A detailed description of such models can be found in [135]. Overall, computational modeling approaches can be useful understanding of the mechanisms of clinically used therapies as well as predicting synergy of novel drug combinations to facilitate design of nanoparticle drug carriers.

Such advances in synergistic drug combinations would be a valuable tool in precision medicine in ovarian cancer in which the molecular profile of a patient’s cancer is used to design a targeted, personalized, and efficacious treatment plan [139]. To accelerate clinical translation of these treatments drug response assays that use patient derived tumor cells are important to developing effective treatments. Organoids, i.e., 3D multicellular aggregates used to model human organ development, derived from primary tumors may be an especially useful platform for personalized medicine as genomic alterations are thought to be recapitulated in the organoid cultures [140]. Such genomic information can guide treatment [139,141,142]. Treatments can be further personalized by combining chemotherapies with immunotherapy treatments based on the tumor biology and the characteristics of the tumor microenvironment [143]. Studying drug-drug interactions at the level of the target, pathway, process, and organism (e.g., using organoids as primary models) is a promising approach to discover effective and translatable combination therapies [144].

Nanoparticle delivery of such drug combinations is an exciting approach to enhance the efficacy of therapy and minimize side effects [139,143]. Precision medicine can also be used to identify receptors for active targeting e.g., folate and human epidermal growth factor receptors [139]. Nanoparticles with folic acid conjugated to the surface have shown enhanced cytotoxic activity compared to non-targeted particles in human ovarian cancer cells (Ovcar-5) [145]. Nanoparticles functionalized with poly-L-aspartate, poly-L-glutamate, and hyaluronate-coated nanoparticles demonstrated highly specific association with ovarian cancer tissue (using patient derived ovarian cancer spheroids) compared to conventional PEGylated nanoparticles [19]. In active targeting, the surface of the nanoparticle is functionalized with one or more targeting moieties to interact specifically with antigens or receptors that are uniquely expressed or overexpressed on the tumor cells compared to normal tissues. By targeting internalizing receptors, this approach can facilitate transport of the nanoparticle into the cell. Ligands targeting intravascular tumor cells or endothelial cells of tumor blood vessels can also be leveraged to further promote accumulation of the nanoparticle within the disease site [146]. Overall, active targeting nanoparticle formulations of chemotherapeutic drugs improves selectivity of cellular uptake and/or cytotoxicity and enhances therapeutic efficacy and safety [145]. Typical targeting moieties include small molecules, polypeptides, nucleic acid aptamers, and antibodies/antibody-fragments. Hyaluronic acid, a glycosaminoglycan, is a polymer that has affinity to the CD44 receptors has also been used as a targeting moiety [145,146]. Targeting strategies that leverage the tumor microenvironment (e.g., pH-sensitive polymers, reversible ligand shielding) has also been considered [147,148]. Overall, co-delivery of chemotherapy with targeting agents in ovarian cancer is an emerging area [32]. Approaches that account for the heterogeneity of patient tumors (e.g., genetic profiles, tumor pathophysiology) in parallel with synergistic drug combinations and active targeting, may lead to improved outcomes. Regulatory aspects and cost-effectiveness of such formulations would need to be carefully considered [18,147,149,150,151].

## 5. Conclusions

Treating ovarian cancer remains challenging with the currently available chemotherapeutic agents (commonly platinum based agents and formulations of taxane). With chemotherapy, relapse can occur due to development of drug resistance. Ovarian cancer cells are known to form resistance to a variety of drugs including cisplatin, carboplatin, and paclitaxel. Combination chemotherapy can improve treatment efficacy (indicated by synergistic effects) and lower systemic toxicity. Selection of the drug combination can target multiple pathways to induce cell death to overcome drug resistance mechanisms. The sequence of delivery of the drug combination and dosing schedule can further improve treatment efficacy. Formulation of drug combinations into nanoparticles can facilitate sustained release to further enhance treatment efficacy. Due to their versatility, polymer-based nanoparticles are an especially promising tool for clinical translation of combination therapies with tunable dosing schedules. Results from this area are encouraging as nanoparticle formulations have been found to increase cytotoxicity in vitro and reduce tumor volume in vivo compared to free drug formulations.

Design of future nanoparticle formulations relies on synergistic drug combination and dosing schedule. Drug synergy is dependent on cell type due to the expression levels of key proteins involved in drug resistance, antiapoptotic, and angiogenesis mechanisms. Thus, computational models to predict synergistic drug combinations and dosing schedules would be a powerful approach to accelerate design of nanomedicines that could improve treatment of ovarian cancer. When combined with genomic information, these formulations may be a promising tool in precision medicine.

## Figures and Tables

**Table 2:** Polymer nanocarrier formulations of paclitaxel-based drug combinations.

Nanoparticle	Drugs	In Vitro	Key Results In Vitro	In Vivo	Key Results In Vivo	Source
Cyclodextrin nanocarries	paclitaxel/curcumin	A2780, SKOV-3	Syngeistic (CI ~ 0.65) when compared to free drugs (CI ~ 1)	-	-	[85]
PEI-g-stearic acid micelles coated with hyaluronic acid	paclitaxel/curcumin	SKOV-3 and SKOV-3-TR30 (multi-drug resistant)	17.3-fold lower IC50 in SKOV-3 cells and 115-fold lower in SKOV-3-TR30 cells compared to free paclitaxel	every other day for 5 times via tail vein injection	Reduces tumor volume compared to free drug (t-test, 5%) and PTX only nanoparticles (*t*-test 10%)	[86]
PEO-PCL nanoparticles	paclitaxel/tamoxifen	SKOV-3, SKOV-3TR	10-fold decrease in IC50 of paclitaxel (SKOV-3), CI ~ 0.4 and (CI ~ 0.7) in SKOV-3TR	SKOV-3, SKOV-3TR xenograft (flank) treated at day 1 and day 24 through tail vein injection	suppressed tumor growth, lowering systemic toxicity, tamoxifen enhanced cytotoxicity of paclitaxel	[87]
mPEG-PCL polymer micelles	paclitaxel/tacrolimus (FK506)	A2780/T (PTX resistant)	5.3-fold decrease in IC50 compared to PTX only micelles;	-	-	[88]
Chitosan/alginate nanocapsules	paclitaxel/lapatnib	OVCAR-3	Increased cytotoxicity compared to PTX	-	-	[89]
PS-PEG nanoparticles	paclitaxel/lapatinib	OVCA-432	1500-fold decrease in IC compared to free drug; co-loaded formulation 4.4 fold decrease in IC50 concentration compared to PTX only formulation; *CI* 0.23; co-loaded formulation more potent than two single drug loaded nanoparticle (*CI* 0.40)	-	-	[90]
EGFR-peptide-PCL nanoparticles	paclitaxel/lonidamine	SKOV-3, SKOV-3TR, OVCAR-5 (MDR)	2-fold decrease in IC50 of paclitaxel in OVCAR-5 cells under hypoxic conditions (no change in IC50 under noroxative conditions or other cell types)	-	-	[91]
PEG-b-PCL micelles	paclitaxel/cyclopamine/gossypol	SKOV-3, ES-2	2D model: no increased potency compared to paclitaxel micelles; 3D model: disaggregation of the spheroid	ES-2, SKOV-3 xenografts via IP injection once a week for 3 weeks via IP injection	significantly reduced tumor volume and extended survival time compared to free paclitaxel	[92]

**Table 3:** Formulations of other drug combinations using polymer micelles/nanoparticles.

Nanoparticle	Drugs	In Vitro	Key Results In Vitro	In Vivo	Key Results In Vivo	Source
Folate-PEG-PLGAnanoparticles	docetaxel/gemcitabine	SKOV-3	3.59-fold drop in the IC50 and improve cytotoxicity in SKOV-3 cells as compared to free drug combination	SKOV-3 xenograft treated every 2 days for 3 weeks via tail vein injections	Reduced tumor volume and rate of tumor growth compared to free drug combination with no organ toxicity	[93]
mPEG-PLA polymer micelles	doxorubicin/gemcitabine	SKOV-3	drug internalization via endocytosis	-	-	[94]
mPEG-PLA nanoparticles	doxorubicin/verapamil	A2780, SKOV-3, A2780/DOX, and SKOV-3/DOXR	micelles increased drug accumulation and enhanced apoptosis	A2780/DOXR and SKOV-3/DOXR xenograft treated every 3 days for 2 weeks via tail vein injection	inhibited tumor growth and increased survival time compared to free doxobucin with reduced side effects	[95]
mPEG-b-poly[N-2-hydroxyethyl)-aspartamide]/phenylboronic acid	Doxorubicin/irinotecan	SKOV-3	Micelles increase IC50 compared to free drug; co-loaded micelles synergistic compared to single drug loaded (*CI* 0.3)	-	-	[96]
Pluronic^®^ F-127 micelles	resveratrol co-loaded with quercetin or curcumin in NPs with free adriamycrin	ES2-Luc, A2780, and A2780ADR, ES2-Luc	Up to 10 –fold reduction in IC50 and synergistic (*CI* < 0.5) in A2780 and A2780ADR cells	ES2-Luc and A2780ADR xenografts treated with weekly injections for 4 weeks via tail vein injection	Significant tumor reduction and cardioprotective effect compared to ADR alone	[97]
mPEG-b-PLA micelles	Chetomin/Everolimus	ES-2, OVCAR-3, TOV-21G	Combination index for co-loaded micelle was <1 compared to single drug loaded micelles	ES-2 treated with weekly injections for 3 weeks via tail vein injection	Significant tumor reduction compared to empty micelles and saline control	[98]
Amphiphilic drug-drug conjugate nanopartpices	floxuridine-chlorambucil	OVCAR-3	Combination index was nanodrugs~0.3 compared to~0.7 for the free drug	-	-	[99]
